# Chemistry, lung toxicity and mutagenicity of burn pit smoke-related particulate matter

**DOI:** 10.1186/s12989-021-00435-w

**Published:** 2021-12-16

**Authors:** Yong Ho Kim, Sarah H. Warren, Ingeborg Kooter, Wanda C. Williams, Ingrid J. George, Samuel A. Vance, Michael D. Hays, Mark A. Higuchi, Stephen H. Gavett, David M. DeMarini, Ilona Jaspers, M. Ian Gilmour

**Affiliations:** 1 Center for Environmental Medicine, Asthma and Lung Biology, University of North Carolina, Chapel Hill, NC 27599 USA; 2 Public Health and Integrated Toxicology Division, Center for Public Health and Environmental Assessment, U.S. Environmental Protection Agency, Research Triangle Park, NC 27711 USA; 3 Biomolecular and Computational Toxicology Division, Center for Computational Toxicology and Exposure, U.S. Environmental Protection Agency, Research Triangle Park, NC 27711 USA; 4 Department of Circular Economy and the Environment, The Netherlands Organisation of Applied Sciences, TNO, Utrecht, The Netherlands; 5 Air Methods and Characterization Division, Center for Environmental Measurements and Modeling, U.S. Environmental Protection Agency, Research Triangle Park, NC 27711 USA; 6 Oak Ridge Institute for Science and Education, Research Triangle Park, NC 27711 USA; 7 Department of Pediatrics, Department of Microbiology and Immunology, and Department of Environmental Sciences and Engineering, University of North Carolina, Chapel Hill, NC 27599 USA

**Keywords:** Military burn pit smoke, Inhalation, Lung toxicity, Mutagenicity, Particulate matter

## Abstract

**Background:**

Open burning of anthropogenic sources can release hazardous emissions and has been associated with increased prevalence of cardiopulmonary health outcomes. Exposure to smoke emitted from burn pits in military bases has been linked with respiratory illness among military and civilian personnel returning from war zones. Although the composition of the materials being burned is well studied, the resulting chemistry and potential toxicity of the emissions are not.

**Methods:**

Smoke emission condensates from either flaming or smoldering combustion of five different types of burn pit-related waste: cardboard; plywood; plastic; mixture; and mixture/diesel, were obtained from a laboratory-scale furnace coupled to a multistage cryotrap system. The primary emissions and smoke condensates were analyzed for a standardized suite of chemical species, and the condensates were studied for pulmonary toxicity in female CD-1 mice and mutagenic activity in *Salmonella* (Ames) mutagenicity assay using the frameshift strain TA98 and the base-substitution strain TA100 with and without metabolic activation (S9 from rat liver).

**Results:**

Most of the particles in the smoke emitted from flaming and smoldering combustion were less than 2.5 µm in diameter. Burning of plastic containing wastes (plastic, mixture, or mixture/diesel) emitted larger amounts of particulate matter (PM) compared to other types of waste. On an equal mass basis, the smoke PM from flaming combustion of plastic containing wastes caused more inflammation and lung injury and was more mutagenic than other samples, and the biological responses were associated with elevated polycyclic aromatic hydrocarbon levels.

**Conclusions:**

This study suggests that adverse health effects of burn pit smoke exposure vary depending on waste type and combustion temperature; however, burning plastic at high temperature was the most significant contributor to the toxicity outcomes. These findings will provide a better understanding of the complex chemical and combustion temperature factors that determine toxicity of burn pit smoke and its potential health risks at military bases.

**Supplementary Information:**

The online version contains supplementary material available at 10.1186/s12989-021-00435-w.

## Background

The World Health Organization (WHO) has reported that heart and lung disease, stroke, and cancers are among the top five causes of global mortality, and one-quarter to one-third of deaths from these diseases are associated with increased exposure to combustion-related air pollution [1]. Moreover smoke emissions from anthropogenic sources (e.g., man-made materials, solid wastes, and construction materials) contain more toxic chemicals than biogenic emissions (e.g., biomass/wildfire smoke) [2, 3], and are either released accidentally from wildland-urban interface (WUI) area wildfires or deliberately by burning household waste and other materials.

Burn pits are a common way to eliminate military waste in the absence of standard waste management systems in war zones. These open burn pits are often located next to military bases, may operate 24 h a day, 7 days a week, and emit many potentially toxic compounds in the air. Often, diesel or jet fuel is used to start or accelerate the fire [4–6]. Most burn pits in Iraq and Afghanistan were permanently shut down in 2009, but some still operate elsewhere [7, 8]. Because over one million U.S. military personnel have been deployed in war zones for the past 20 years [9], military communities and public health professionals have become increasingly concerned about exposures to burn pit smoke. There is growing concern that a significant number of Iraq and Afghanistan war veterans continue to experience health problems associated with exposures to a variety of airborne hazards during military service [4–6, 10–20].

While various sources, such as geologic dust, vehicle exhaust, and ordnance, contribute to ambient particulate matter (PM) at U.S. military sites in war zones, especially in the Middle East, one report suggested that smoke emitted from burn pits is a major source of air pollution [21]. Additionally, associations between burn pit emissions and respiratory symptoms, including asthma [6], respiratory symptoms [20], acute eosinophilic pneumonia [19], and constrictive bronchitis [13], have been reported, whereas other studies have not identified significant effects [22–27]. What is clear is that smoke from burn pits contains numerous potentially toxic compounds, including polycyclic aromatic hydrocarbons (PAHs), volatile organic compounds (VOCs), and heavy metals [7, 28–33]. However, the role of specific smoke components on disease incidence or severity following exposure is not well understood [28–31].

A challenge with laboratory-based toxicity studies of burn pit smoke is to first generate relevant representative test samples and then simulate exposures using in vitro or in vivo models. Many studies have successfully generated combustion byproducts from various sources [28–30, 34–48], but little information is available on the toxicity of the combustion smoke emissions. Previously, we developed an automated furnace connected to a cryotrap system that can control combustion phases (smoldering and flaming), generate combustion emissions from different fuels with reproducible physico-chemical characteristics, and collect large quantities of smoke condensates for subsequent chemical analysis and toxicity testing [49].

Here we utilized this approach to simulate military burn pit smoke emissions from representative fuel samples under two distinct burning conditions and sought to identify components of the burn pit condensates associated with toxicity outcomes. Condensates were obtained from a combination of three different types of burn pit materials (wood, paper, and plastic) either alone or in a prescribed combination with and without diesel fuel as an accelerant under two different combustion conditions (smoldering and flaming). We assessed the condensates for potential to cause lung toxicity in mice after oropharyngeal aspiration, and for mutagenicity in the *Salmonella* (Ames) mutagenicity assay. We also calculated mutagenicity emission factors and compared them to published data for other combustion emissions, such as biomass smoke, cookstove emissions, diesel exhaust, and waste incineration.

## Results

### Physico-chemical characteristics of the burn pit smoke

**Fig. 1:**
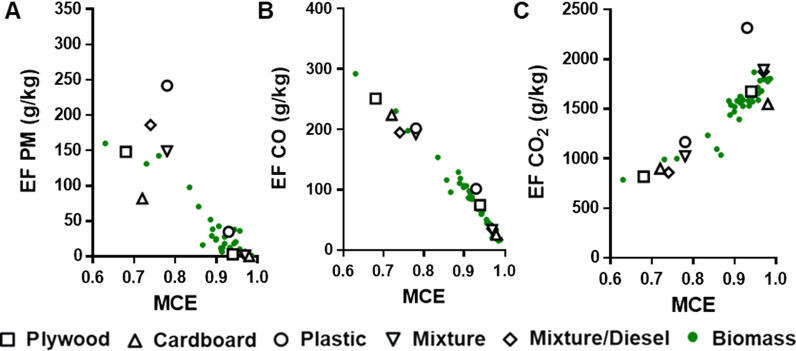
Comparison of emission factors (EFs) of the burn pit smoke and published EFs from various biomass combustions. **A** EF for PM, **B** EF for CO, and **C** EF for CO_2_ vs. modified combustion efficiency (MCE). EFs from biomass combustions are obtained from published studies [44, 49]

Military waste burn pit smoke emissions were generated from five waste types (hereinafter designated plywood, cardboard, plastic, mixture, and mixture/diesel) under two combustion phases (smoldering and flaming); their smoke characteristics are summarized in Table 1. Average PM emission factors (EFs) of the burn pit smoke were 161.5 g/kg during the smoldering combustion (0.74 of modified combustion efficiency) and 8.3 g/kg during the flaming combustion (0.96 of modified combustion efficiency). Notably, the largest contributor to PM emissions from the flaming combustion was the plastic smoke, which contained approximately 20 times higher PM mass than other (flaming) burn pit smoke emissions. This emission characteristic of the plastic smoke also showed distinct differences for PM and CO_2_ EFs compared to biomass smoke emissions under similar combustion conditions (Fig. 1). The flaming plastic smoke contained up to approximately 10 times higher CH_4_ mass than other flaming burn pit smoke emissions. The plastic smoke PM had 64 and 74% total carbon for smoldering and flaming conditions, respectively, whereas other smoke PM averaged 50% total carbon for both combustion conditions. The same combustion condition (flaming or smoldering) produced a similar NOx level, but approximately 5 times higher levels were measured in flaming (3.2 g/kg) than smoldering (0.6 g/kg) phases. Mass median aerodynamic diameters of PM in the burn pit smoke ranged from 0.3 to 2.8 µm for all tested waste smoke emissions. In general, the particles from flaming plywood and cardboard and the mixture were smaller than the other fuels and conditions.

**Table 1:** Characteristics of the burn pit smoke

Variable	Plywood		Cardboard		Plastic		Mixture		Mixture/diesel	
	Smolder	Flame	Smolder	Flame	Smolder	Flame	Smolder	Flame	Smolder	Flame
MCE^a^	0.68	0.94	0.72	0.98	0.78	0.93	0.78	0.97	0.74	0.97
CO (g/kg)^b^	250.6	74.5	223.6	25.5	202.1	101.9	191.5	33.9	195.0	36.2
CO_2_ (g/kg)^b^	818.2	1674.1	898.7	1547.8	1166.3	2315.7	1022.6	1891.9	860.2	1873.8
PM (g/kg)^b^	148.3	2.6	81.6	0.4	242.3	34.9	149.4	1.3	186.1	2.2
CH_4_ (g/kg)^b^	16.4	5.6	9.6	0.8	5.3	7.5	4.8	0.7	6.7	1.7
VOCs (g/kg)^b^	48.0	27.0	26.0	4.0	544	136.0	127.0	17.0	164.0	29.0
NOx (g/kg)^c^	1.5	3.8	0.4	2.9	0.2	2.4	0.4	3.9	0.3	2.9
OC (% of PM mass)	45.5	45.5	41.3	36.1	64.3	68.8	55.5	48.4	58.4	60.0
EC (% of PM mas)	0.4	0.4	0.4	1.6	0.1	5.6	0.2	5.6	0.1	2.3
EOM (% of PM mass)	64	59	61	38	71	39	70	49	71	65
PM Size (µm)^d^	1.5 [1.5]	0.3 [1.1]	1.0 [1.2]	0.3 [1.1]	1.3 [1.4]	2.8 [2.0]	1.5 [1.4]	0.3 [1.2]	1.6 [1.3]	2.2 [2.0]

^a^Modified combustion efficiency (MCE) = ΔCO_2_/(ΔCO_2_ + ΔCO)

^b^Emission factor (EF) *t* (g/kg) = (fuel carbon fraction x mass of carbon emitted as *t* x molecular weight *t* × 1000) / (molecular weight carbon x total mass of carbon)

^c^Emission factor (EF) NOx (g/kg) = (mass of NOx / total mass of carbon emitted) x (mass of carbon in the fuel / total mass of fuel)

^d^Mass median aerodynamic diameter (MMAD) of PM; values in brackets represent the geometric standard deviation

**Fig. 2:**
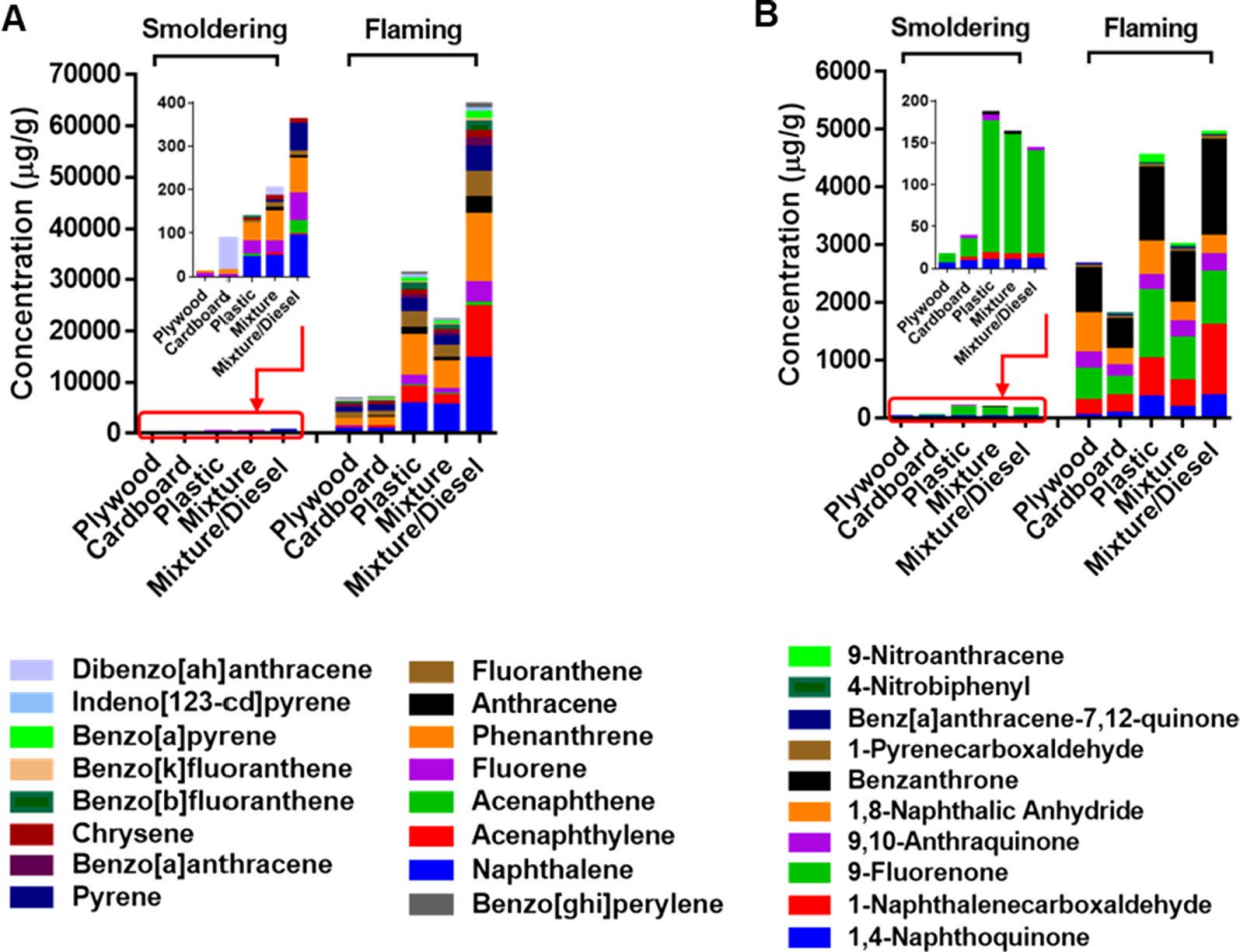
Concentrations of PAHs in the burn pit smoke PM emitted from different waste types and combustion phases*.* **A** 16 EPA priority PAHs and **B** Nitro- and oxy-PAHs

**Fig. 3:**
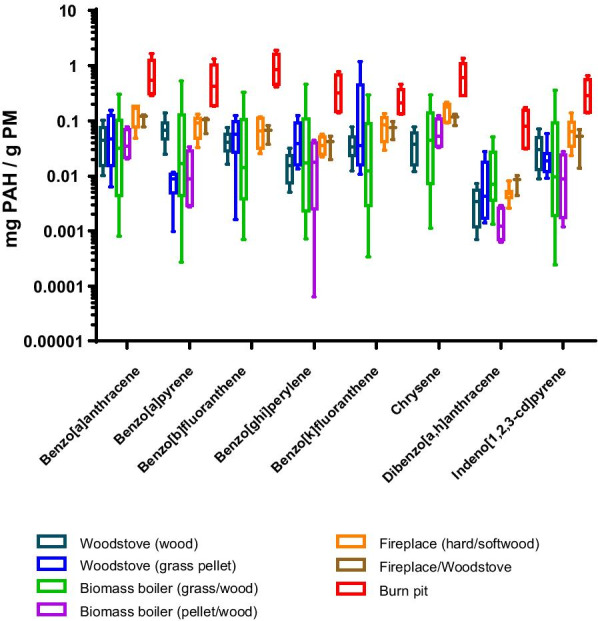
Comparison of particle-bound PAH concentrations in the burn pit smoke and various biomass smoke emissions*.* PAH concentrations in PM emitted from biomass combustions are obtained from published studies: wood smoke from woodstove [39], grass pellet smoke from woodstove [101], grass/wood smoke from biomass boiler [38], wood/pellet smoke from biomass boiler [47], hard/softwood smoke from fireplace [35], biomass smoke from fireplace/woodstove [30]

The PAH composition in the PM from the burn pit smoke emissions are given in Fig. 2a and Additional file 1: Table S2. PAH levels in the flaming smoke PM were at least an order of magnitude higher than those of the smoldering burn pit smoke PM and were appreciably more abundant than those found in various biomass smoke emissions published in the literature (Fig. 3). The sum of EPA priority PAHs in the PM condensates from flaming combustion was greatest for the mixture/diesel waste (64 mg PAH/g PM), followed by the plastic (31 mg PAH/g PM), the mixture (22 mg PAH/g PM), the cardboard (7 mg PAH/g PM), and the plywood waste (6 mg PAH/g PM). The maximum concentration distribution of individual PAHs in the PM emissions varied with the waste burned. Generally, phenanthrene, naphthalene, and fluoranthene were the predominant compounds measured in the condensates during flaming combustion. Additionally, relatively high levels of acenaphthylene were noted in the plastic and mixture/diesel burns. Similar to the PAH concentrations, higher levels (> 20 times) of nitrated/oxygenated (nitro-/oxy-) PAHs were also measured in the flaming smoke PM (Fig. 2b and Additional file 1: Table S2) in the order: mixture/diesel ≥ plastic > mixture ≥ plywood > cardboard. Major components were naphthalenecarboxaldehyde, benzanthrone, and fluorenone, which accounted for 60 – 80% of total nitro- and oxy-PAHs from the flaming smoke PM.

The sums of speciated volatile organic compound (VOC) emissions measured in the actual smoke for each waste and combustion phase are summarized in Table 1. These measurements indicate that VOC emissions were consistently greater under smoldering conditions compared to flaming conditions for the same waste type by approximately 2–7 times. Regardless of combustion phase, the highest sum of VOC emissions was observed from the plastic burning, whereas the lowest values were measured for the cardboard. The relative contributions of the most abundant VOCs measured in the burn pit smoke for each waste and combustion phase are shown in Additional file 1: Fig. S1. The largest contributors to VOC emissions were air toxics, such as aromatics, unsaturated hydrocarbons, and aldehydes, which are typically associated with combustion emissions. Notable differences in the VOC chemical profiles were observed between flaming and smoldering phases for all wastes. For example, benzene was the dominant species in all flaming emission profiles, representing from 38 to 55% of the total speciated VOC mass. The chemical composition of VOC emissions in the smoldering phase varied with the starting fuel. The cardboard and plywood smoldering emissions were dominated by carbonyl compounds, whereas the plastic, mixture and mixture/diesel emissions in the smoldering phase had the largest relative contribution from styrene. The influence of diesel in the mixture/diesel emissions is indicated by the presence of C9–C12 alkane species (e.g., nonane, decane and dodecane) in the flaming and smoldering phases.

### Lung toxicity and physiology of the burn pit smoke PM

**Fig. 4:**
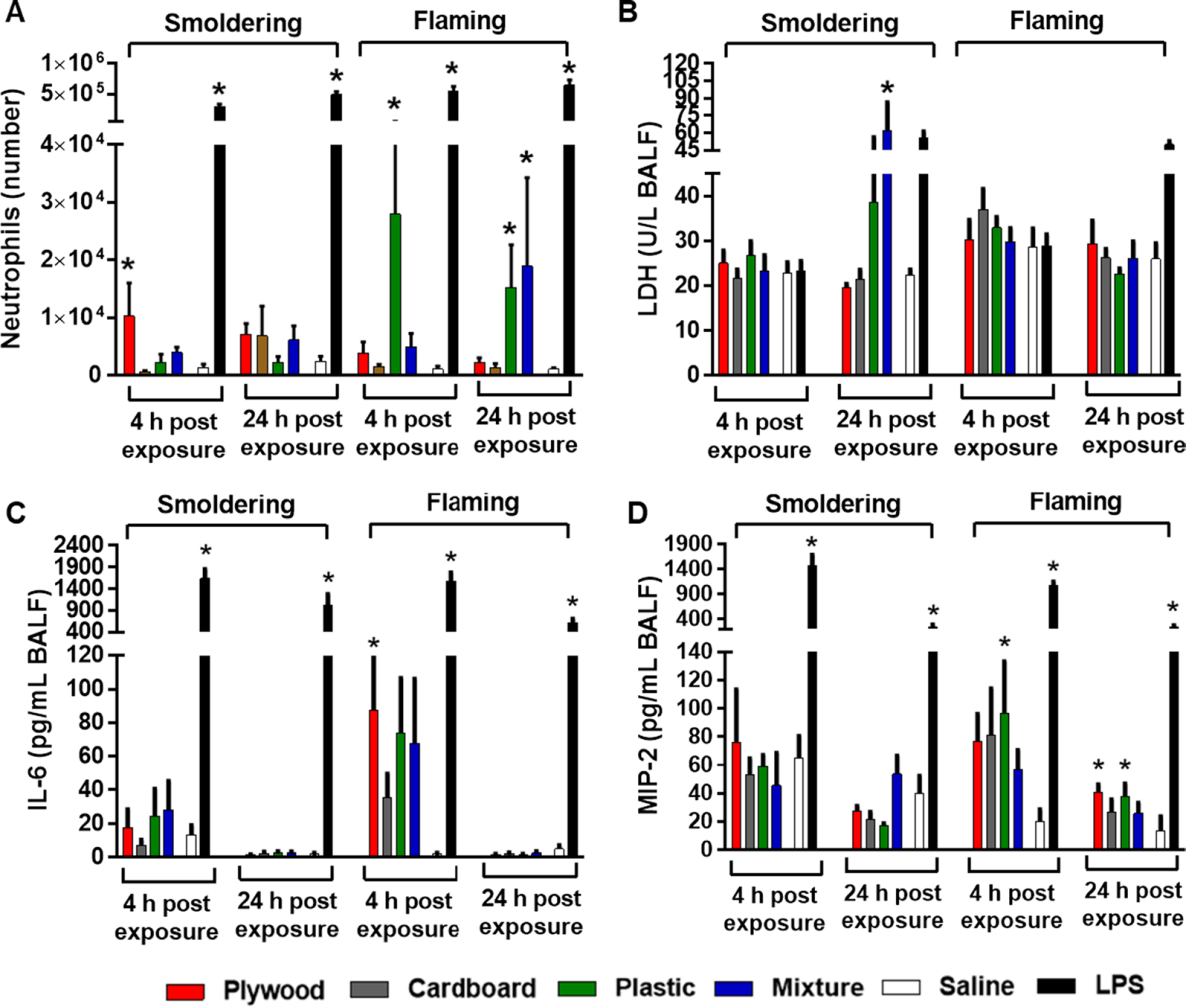
Lung toxicity of the burn pit smoke PM emitted from different waste types and combustion phases. **A** neutrophil response, **B** LDH response, **C** interleukin (IL)-6 response, and **D** macrophage inflammatory protein (MIP)-2 response. Mice were exposed to the PM (100 µg) by oropharyngeal aspiration and BALF was obtained at 4 and 24 h post exposure. Data are mean ± SEM obtained from 6 mice for each group. *P < 0.05 compared with the saline-exposed (a negative control) group from the same time point. Mice exposed to 2 µg of lipopolysaccharide (LPS) served as a positive control

At 4 and 24 h after exposure by oropharyngeal aspiration, bronchoalveolar lavage fluid (BALF) of the mice was analyzed for lung cell injury and inflammation. The smoldering smoke samples resulted in small increases in neutrophils at 4 and 24 h (Fig. 4a). Of these responses only the plywood smoke PM reached significance at 4 h post-exposure. In contrast the flaming plastic smoke condensates caused significant increases in neutrophils at both 4 and 24 h, with the flaming mixture smoke having a persistent significant increase at 24 h post-exposure. Lactate dehydrogenase (LDH) levels did not differ among the various exposures apart from the smoldering mixture condensate, which had significant increases at 24 h post-exposure (Fig. 4b). Pro-inflammatory cytokines, which typically track as early signals of neutrophil infiltration, were also increased after exposures to the flaming smoke condensate but not the smoldering smoke PM (Fig. 4c, d). Interleukin-6 (IL-6) was increased for all the flaming samples at 4 h post-exposure, although only significantly for the plywood smoke PM. The levels decreased to around the limit of detection by 24 h. Macrophage inflammatory protein-2 (MIP-2) levels were increased at 4 h post-exposures to all the flaming smoke PM, with significance achieved only with the plastic smoke PM. Although MIP-2 levels were lower at 24 h, the values were significantly different from the saline vehicle for the plywood and plastic smoke PM. Tumor necrosis factor-α (TNF-α) was not elicited to any great extent except for the LPS positive control (data not shown). None of the smoke PM exposures significantly altered any of the hematological parameters studied (data not shown).

**Fig. 5:**
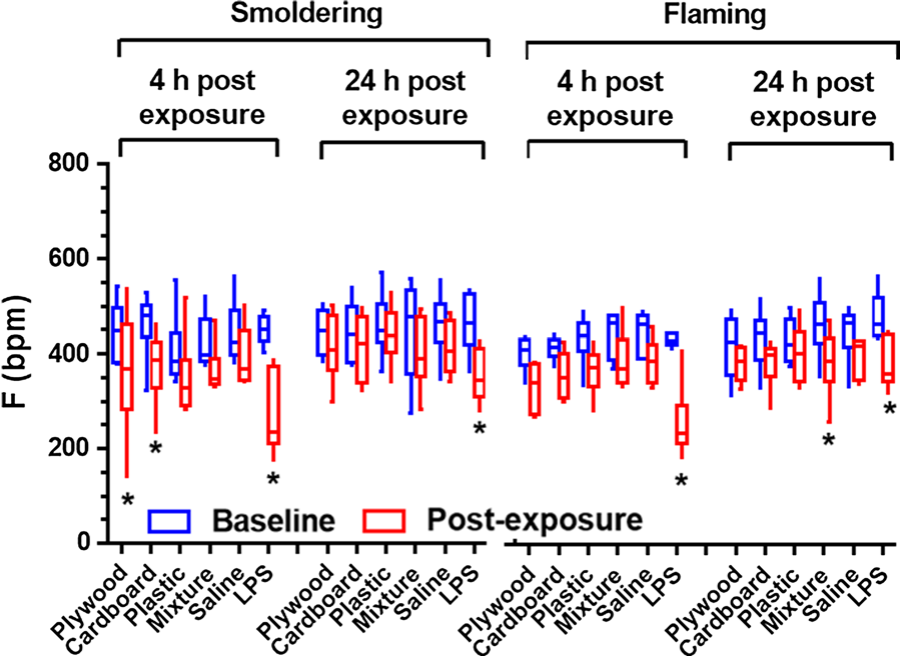
Breathing frequency of mice exposed to the burn pit smoke PM emitted from different waste types and combustion phases*.* Data are mean ± SEM obtained from 6 mice for each group. *P < 0.05 compared with the pre-exposed (baseline) group from the same time point

Changes in respiratory parameters (Te, Ti, PIF, PEF, RT, MV, TV, and F) in mice were monitored prior to the burn pit smoke exposure (baseline), and at 4 and 24 h post-exposure (prior to necropsy). Decreases in breathing frequency (F) were detected at 4 h after exposure to the smoldering plywood and carboard smoke condensates (Fig. 5). These changes were not persistent at 24 h post-exposure. No flaming PM exposed mice had changes in F at 4 h; however, the mixture smoke condensate caused a significant decline in F at 24 h post-exposure. The LPS-exposed mice had significant declines in F at both time points. The smoldering cardboard condensate produced a significant increase in inspiratory time (Ti) at 4 h post-exposure, whereas the smoldering plywood sample produced an increased expiratory time (Te) at 4 h (Additional file 1: Table S3). No significant changes in respiratory flows (PIF and PEF), volumes (TV and MV) or relaxation time (RT) were noted other than a significant reduction in MV for LPS at 4 h for both studies (Additional file 1: Table S3).

### Mutagenicity of the burn pit smoke PM

**Fig. 6:**
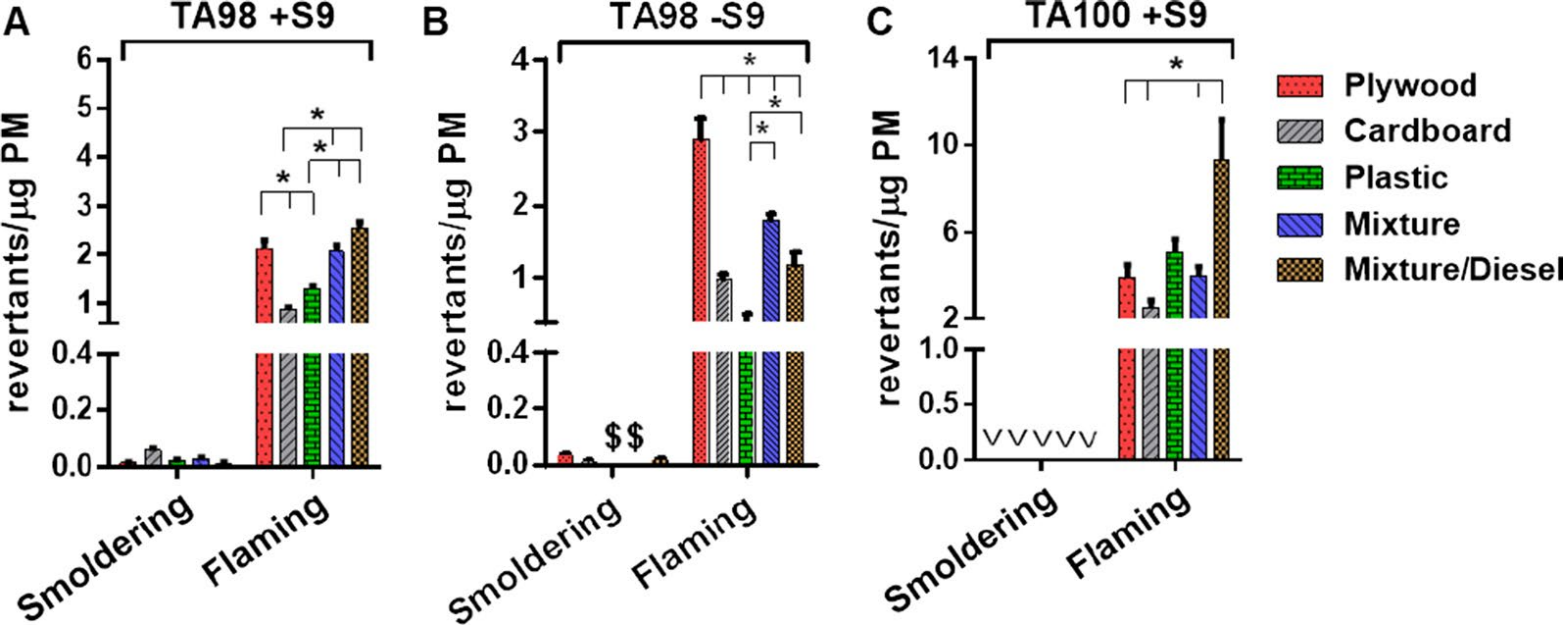
Mutagenicity of the burn pit smoke PM in the TA98 -S9 and TA100 + S9 *Salmonella* strains*.* **A** TA98 + S9, **B** TA98 -S9, and **C** TA100 + S9. Mutagenic potencies (rev/µg PM) of the extractable organic material (EOM) were calculated from the slope of the linear portion of the dose–response curve created by the average of the primary data (rev/plate) from two to four independent mutagenicity experiments (Additional file 1: Figs. S2 and S3). The mutagenic potencies of the EOM were then multiplied by the percent EOM to give mutagenic potencies of the PM (rev/µg PM). Data are presented as mean ± SEM. *These values were statistically significant (*p* < 0.05). ^$^Samples with slopes with trend tests with *p* > 0.05 were considered non-mutagenic and given a mutagenic potency of zero. ^v^Insufficient sample available to generate dose–response curves; samples were not mutagenic at 1000 µg EOM/plate

The extractable organic materials (EOMs) from the flaming smoke PM were mutagenic in all three strains and S9 conditions; however, none of the EOMs from the smoldering samples were mutagenic in TA100 nor were those from the smoldering plastic or mixture smoke PM in TA98 -S9 (Fig. 6 and Table 2). The mutagenic potencies of the flaming smoke EOM (rev/µg EOM) and PM (rev/µg PM) in all strains and S9 conditions were greater than those of their comparable smoldering smoke PM, typically by more than an order of magnitude. Among the flaming smoke PM in strain TA98 + S9, the most potent EOM or PM was the plywood and the mixture/diesel, suggestive of a role for PAHs and aromatic amines that induce frameshift mutations. With respect to the mutagenicity of the flaming smoke PMs in TA98 -S9, the most potent EOM or PM was the plywood, indicating that some portion of its mutagenicity was due to nitroarenes (nitro-PAHs). With respect to the mutagenicity of the flaming smoke PM in TA100 + S9, the most potent EOM or PM was the mixture/diesel, indicating the presence of PAHs. The EOMs and PMs of the flaming smoke were most potent in their mutagenicity in strain TA100 + S9, indicating that PAHs played a predominant role, relative to aromatic amines and nitro-PAHs.

**Table 2:** Mutagenic potencies and mutagenicity EFs of the burn pit smoke PM in *Salmonella* TA98 ± S9 and TA100 + S9

Model outcomes	Plywood		Cardboard		Plastic		Mixture		Mixture/diesel	
	Smoldering	Flaming	Smoldering	Flaming	Smoldering	Flaming	Smoldering	Flaming	Smoldering	Flaming
TA98 + S9										
rev/µg EOM	0.024 ± 0.005	3.610 ± 0.310	0.049 ± 0.012	2.320 ± 0.120	0.030 ± 0.008	3.330 ± 0.140	0.040 ± 0.008	4.220 ± 0.300	0.018 ± 0.005	3.910 ± 0.220
rev/µg PM	0.015 ± 0.003	2.130 ± 0.183	0.030 ± 0.007	0.882 ± 0.046	0.021 ± 0.006	1.299 ± 0.055	0.028 ± 0.006	2.068 ± 0.147	0.013 ± 0.004	2.542 ± 0.143
rev** × **10^5^/kg fuel	23.34 ± 4.86	57.29 ± 4.92	24.81 ± 6.08	3.44 ± 0.18	51.97 ± 13.85	472.72 ± 19.87	42.00 ± 8.40	26.26 ± 1.87	24.03 ± 6.67	55.91 ± 3.15
rev** × **10^5^/MJ_th_	1.17 ± 0.24	2.86 ± 0.25	1.42 ± 0.35	0.20 ± 0.01	1.60 ± 0.43	14.55 ± 0.61	1.86 ± 0.37	1.16 ± 0.08	1.06 ± 0.30	2.47 ± 0.14
TA98 -S9										
rev/µg EOM	0.050 ± 0.010	4.910 ± 0.490	0.021 ± 0.004	2.610 ± 0.150	0.0 ± 0.0^a^	1.050 ± 0.250	0.0 ± 0.0^a^	3.670 ± 0.200	0.024 ± 0.009	1.810 ± 0.250
rev/µg PM	0.032 ± 0.006	2.897 ± 0.289	0.013 ± 0.002	0.992 ± 0.057	0.0 ± 0.0	0.410 ± 0.098	0.0 ± 0.0	1.798 ± 0.098	0.017 ± 0.006	1.177 ± 0.163
rev** × **10^5^/kg fuel	48.64 ± 9.73	77.93 ± 7.78	10.63 ± 2.03	3.87 ± 0.22	0.0 ± 0.0	149.06 ± 35.49	0.0 ± 0.0	22.83 ± 1.24	32.04 ± 12.01	25.88 ± 3.58
rev** × **10^5^/MJ_th_	2.43 ± 0.49	3.90 ± 0.39	0.61 ± 0.12	0.22 ± 0.01	0.0 ± 0.0	4.59 ± 1.09	0.0 ± 0.0	1.01 ± 0.06	1.42 ± 0.53	1.15 ± 0.16
TA100 + S9										
rev/µg EOM	No data^b^	6.580 ± 0.960	No data^b^	6.570 ± 0.890	No data^b^	12.960 ± 1.640	No data^b^	8.130 ± 0.810	No data^b^	14.320 ± 2.820
rev/µg PM	No data^b^	3.882 ± 0.566	No data^b^	2.497 ± 0.338	No data^b^	5.054 ± 0.640	No data^b^	3.984 ± 0.397	No data^b^	9.308 ± 1.833
rev** × **10^5^/kg fuel	No data^b^	104.43 ± 15.23	No data^b^	9.74 ± 1.32	No data^b^	1839.80 ± 232.81	No data^b^	50.59 ± 5.04	No data^b^	186.16 ± 36.66
rev** × **10^5^/MJ_th_	No data^b^	5.22 ± 0.76	No data^b^	0.56 ± 0.08	No data^b^	56.61 ± 7.16	No data^b^	2.24 ± 0.22	No data^b^	8.24 ± 1.62

Mutagenic potencies of the EOM (rev/µg EOM ± SEM) are slopes of linear regressions calculated from the dose–response data (rev/plate) from 4 independent experiments for the smoldering samples (Additional file 1: Fig. S2) and 2 independent experiments for the flaming samples (Additional file 1: Fig. S3). Mutagenic potencies of the PM (rev/µg PM ± SEM) were calculated by multiplying the rev/µg EOM values by the %EOM for each fuel in Table 1. Mutagenicity emission factors (EFs) of the PM (rev/kg fuel) were calculated by multiplying the rev/µg PM values by the PM emission factors for each fuel in Table 1. Mutagenicity EFs of the PM (rev/MJ_th_) were calculated by dividing the rev/kg fuel values by the heat energy (MJ_th_/kg fuel) of each fuel, which was 20.00 for the plywood [98], 17.5 for the cardboard [99], 32.5 for the plastic [100], and 22.6 for the mixture and mixture/diesel [100]

^a^Samples with slopes with trend tests with *P* > 0.05 were considered non-mutagenic and given a mutagenic potency of zero

^b^Insufficient sample available to generate dose–response curves; most samples were negative at 1000 µg EOM/plate

**Fig. 7:**
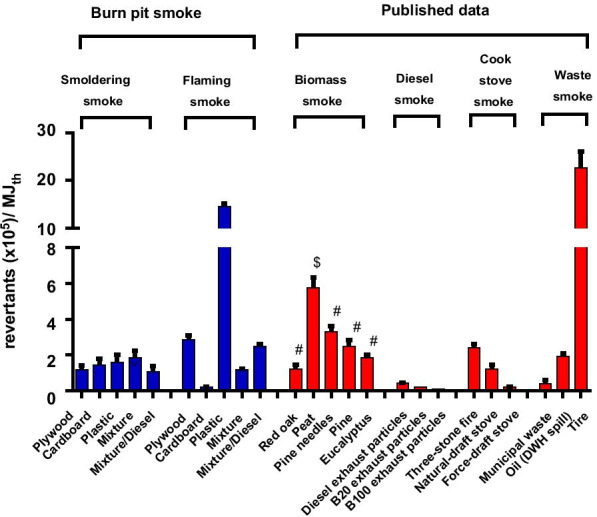
Comparison of mutagenicity EFs of the burn pit smoke and various combustion smoke PMs in strain TA98 + S9. Mutagenicity emission factor (EF) calculated based on the emitted PM mass per mass of fuel burned. Mutagenic potencies of the PM (rev/µg PM; Fig. 6a) were converted to mutagenicity EFs (rev/MJ_th_) using the values for the heat energy of each fuel (MJ_th_/kg fuel). Data are presented as mean ± SEM. ^#^Mutagenicity EFs were estimated based on the assumption that 80% of the emissions were produced by flaming and 20% by smoldering. ^$^Mutagenicity EFs were estimated based on the assumption that 20% of the emissions were produced by flaming and 80% by smoldering. The mutagenicity EFs for biomass smoke were 1.2, 5.8, 3.3, 2.5, and 1.9 × 10^5^ rev/MJ_th_ for the red oak, peat, pine needles, pine and eucalyptus, respectively [49]. The mutagenicity EFs for diesel smoke were 0.4, 0.2, and 0.1 × 10^5^ rev/MJ_th_ for the diesel exhaust particles, B20 exhaust particles, and B100 exhaust particles, respectively [46]. The mutagenicity EFs for cookstove smoke were 2.4, 1.2, and 0.2 × 10^5^ rev/MJ_th_ for the three-stone fire, natural-draft stove, and force-draft stove, respectively [45]. The mutagenicity EFs for waste smoke were 0.4, 1.9, and 22.7 × 10^5^ rev/MJ_th_ for the municipal waste [82], oil [102], and tire [79], respectively

In TA98 + S9, the mutagenicity EFs (rev/kg fuel or rev/MJ_th_) of flaming smoke PM were greater than those of smoldering samples for the plywood, the plastic, and the mixture/diesel smoke PM, whereas the opposite was the case for the cardboard and the mixture smoke PM (Fig. 7 and Table 2). In TA98 -S9, the mutagenicity EFs of the flaming smoke PM were also greater for the plywood and plastic as well as the mixture smoke PM, whereas the opposite was the case for the cardboard and mixture/diesel smoke PM (Table 2). Thus, the mutagenicity EFs were consistently greater for flaming than for smoldering samples for the plywood and the plastic smoke PM in TA98 regardless of S9, indicating that PAHs, nitro-PAHs, and aromatic amines played a greater role in the flaming rather than smoldering mutagenicity EFs of these samples. In contrast, these chemical classes played a dominant role in the mutagenicity EFs of the smoldering cardboard smoke PM.

The mutagenicity EFs for the flaming smoke PM were greatest in TA100 + S9 (Table 2), indicating that PAHs played a predominant role in the mutagenicity EFs of the flaming smoke samples. Although the smoldering smoke PM was not mutagenic in TA100, the mutagenicity EFs of smoldering samples of the cardboard, the plastic, and the mixture were greatest in TA98 + S9, indicating a role for some PAHs and aromatic amines, whereas those of the plywood and the mixture/diesel smoke PM were greatest in TA98 -S9, indicating a role for nitro-PAHs. Among the flaming samples, the mutagenicity EFs of the plastic smoke PM were the highest in all three strains and S9 conditions, whereas those of the cardboard smoke PM were the lowest. Among the smoldering smoke samples, the mutagenicity EFs were not significantly different.

## Discussion

### Burn pit-related smoke emissions from a lab-scale combustion system

Woods, plastics, and papers are the most prevalent components of military waste that were commonly incinerated in burn pits during the Iraq and Afghanistan wars, making up about 61% (by weight) of the average waste sites [28, 33, 50]. Interestingly, these same components are also the major portion of municipal solid waste materials in the United States [50], suggesting that the smoke emissions presented here could represent combustion byproducts from not only military burn pits but also any fire incidents associated with burning the solid portion of waste generated by households, and may have relevance to anthropogenic smoke that occurs during wildfire events at the wildland urban interface (WUI). Here we burned five different waste materials (plywood, cardboard, plastic, mixture, and mixture/diesel) representative of emissions from military burn pits. We used an automated combustion system to generate well-defined and reproducible burn pit smoke emissions. Because combustion smoke is a complex and dynamic chemical mixture that can change rapidly or stabilize depending on burning conditions [51], better definition of the chemistry of burn pit smoke will help our understanding of complex smoke characteristics and their role in subsequent toxicity outcomes.

We demonstrated previously that characteristics of biomass smoke emissions from our combustion system agreed well with those collected from field and laboratory measurements [49]. Similarly, comparing our emission factor (EF) data to existing values in the literature (Fig. 1) clearly showed that characteristics of smoke from burning the plywood in this study correlated well with published smoke emission data from various biomass combustions. However, emission characteristics from non-wood burn pit materials (i.e., cardboard and plastic) were quite different. For example, burning plastics released higher concentrations of PM, whereas lower concentrations of PM were emitted from cardboard burning when compared to wood combustion. This suggests that major pollutant emissions from burn pits, especially PM and CO_2_, could largely change depending on types of waste burned, and that burning plastic-containing waste will produce considerably more PM than burning wood-based material [52].

We also demonstrated that organic carbon and PAHs were emitted to a greater extent from burning plastic-containing wastes (Fig. 2). Moreover, PAHs with higher vapor pressure accounted for a higher fraction of the PM composition, and PAH levels emitted from flaming combustion were at least an order of magnitude greater than those in the smoldering emissions. This result is consistent with the understanding that highly efficient combustion of biomass and perhaps other materials occurs in the flaming phase, leading to higher emissions of PAH byproducts [43, 53]. Higher temperatures like those observed during the flaming stage are required for de novo PAH formation, which would explain the greater PAH concentrations observed in the flaming condition [38, 54]. The relatively high level of PAH in the mixture/diesel smoke PM is likely associated with the addition of diesel in the burn pit material containing PAH and lighter aromatic subunits that can rearrange to form PAH and further increase flame temperatures to levels optimal for de novo PAH formation [34].

The high PAH concentrations in the flaming burn pit smoke were also confirmed by comparison with those from a series of biomass burning investigations performed using a variety of appliances (Fig. 3). The comparison was constrained to a subset of eight EPA priority PAH compounds with relatively low vapor pressures (mostly particle-bound PAHs), which eliminates any bias in semi-volatile compound reporting across studies due to differing sampling conditions that influence phase partitioning and media-based sample fractions [38]. Our data clearly show that individual PAH concentrations in PM emissions from the burn pit combustions under flaming conditions were approximately an order of magnitude higher than the average of those from wood-burning appliances. These semi-volatile PAHs are of toxicological concern owing to their known mutagenicity and carcinogenicity [55, 56] and are also regarded as hazardous chemicals that are regularly detected in air samples collected at military bases in Iraq [4]. We reported previously that the mutagenic potencies of PM emissions from biomass-burning sources were greater from flaming than smoldering phases on an equal PM mass basis, further justifying a focus on PAH chemistry [49].

The plastic-containing wastes had the highest levels of VOC emissions (Additional file 1: Fig. S1 and Table 1). This is consistent with a larger general trend showing higher levels of VOCs emitted from burning of anthropogenic sources compared to biogenic sources [2, 3]. The most abundant VOC species observed for burning of plastic-containing wastes were styrene and 1-hexene from the smoldering combustions and benzene from the flaming combustions, suggesting that even though similar types of waste are burned, different combustion temperatures produce not only different amounts, but also different types of VOCs [57]. This is also consistent with the finding that most of the non-substituted aromatics (e.g., benzene) were emitted from high temperatures, but multiple substituents (e.g., styrene) arose from low temperature combustion [58]. Although benzene and styrene, which are among the VOCs detected, are toxic [59–63], these vapor phase constituents would not have been captured to any large degree in the cryotrap and would likely not be present to any degree in the condensates. Toxicological assessments following inhalation of these emissions that include the whole smoke including vapor and gas phase constituents are planned in future studies. Here we only focused on particle-enriched condensates to assess the potential toxicity of the burn pit smoke PM.

### Acute lung toxicity caused by the smoke of burning plastic-containing wastes

There is growing concern about potential links between burn pit emissions and subsequent health outcomes among veterans, active service members, and people living near military sites during wars [4–6, 10–20]. The majority of studies, however, rely predominately on self-reported or retrospective surveillance data [5, 14, 24, 25, 64]. No toxicological study has been carried out with combustion byproducts of military burn pits to probe their potential toxicity. We clearly demonstrated that on an equal PM mass exposure basis, the flaming smoke PM had higher lung toxicity than the smoldering samples (Fig. 4). Specifically, the smoke PM from burning of plastic-containing waste (i.e., the plastic and the mixture) were more pro-inflammatory than those from other types of waste. For example, a good correlation between the neutrophil numbers in the lungs and the total sum of PAHs per g PM was observed (Pearson’s *r* = 0.87, Additional file 1: Fig. S4). This is also consistent with our previous research on the correlation between organic matter or PAHs of the biomass smoke PM and their toxicity [49]. Because PAH toxicity is mostly mediated by aryl hydrocarbon receptor (AHR) which is important for cellular function and maintenance of cardiovascular function [65, 66], the health effects of PAHs are not limited to the lungs but also other organs (e.g., heart [67] and brain [68]). Finally, we anticipate that the toxicity of burn pit smoke PM arises from exposures to combinations of chemicals in the smoke [69] although quantitative speciation of toxic chemicals provides important information and correlations with the observed health outcomes.

The flaming plastic smoke PM, caused a significant increase of MIP-2 at 4 h post-exposure compared to other smoke PM and this was associated with a later enhanced neutrophil influx into the lungs [70]. A small but significant increase in IL-6 was observed following exposure to the flaming plywood smoke PM. This is consistent with our previous finding [49] of cytokine levels in mice exposed to the smoke PM from burning of pine wood, which is the most common type of wood used for the manufacture of plywood products. Our findings suggest that this short-term exposure to the flaming burn pit smoke PM induced an acute inflammation (reflected by MIP-2 and IL-6 levels) rather than cellular damage or death (reflected by LDH levels) in the lungs.

Although we produced well-defined and reproducible burn pit smoke to provide accurate toxicological responses, there are several factors that should be addressed when considering real-world exposure situations. The waste components of burn pits presented herein are limited to certain waste types. For example, we burned only non-chlorinated plastics, but other plastics including chlorine-containing materials can produce persistent organic pollutants (e.g., dioxins, furans and polychlorinated biphenyls) which are highly toxic compounds [32, 71] and likely influence biological responses to real world burn pit smoke. Similarly, other materials including clothing, munitions, food scraps, vehicle spare parts, sewage, etc., are also found in burn-pits [4] but were beyond the scope of this laboratory-based simulation. The PM dose used in this study (equivalent to approximately 3 mg/m^3^; see “Methods” section for details) appeared to be lower than several regional PM levels from the military deployment sites in Iraq and Afghanistan [6, 64]. Moreover, we generated fresh burn pit smoke and only tested the toxicity of PM-enriched condensates, but in reality, the freshly emitted smoke may react in the atmosphere through oxidative and photochemical reactions to produce secondary products (aged burn pit smoke), which are more reactive due to generation of oxidative species and other gas and particle phase atmospheric transformation products. For example, we have shown that the simulated production of atmospheric transformation products from various VOCs are more toxic and mutagenic than parent compounds [72, 73]. Thus, real-world exposures to burn pit smoke could induce higher lung toxicity than reported here.

We demonstrated that some respiratory parameters measured across the various burn pit materials and combustion conditions were significantly changed at 4 h post-exposure, but they returned to normal after 24 h, except for the exposure to the flaming mixture smoke PM (Fig. 5). Although contrasting results were observed in lung toxicity testing, lung physiology testing showed relatively low adverse outcomes. Airflow limitation in the lungs is normally associated with inflammatory responses [74, 75], but this may not always be the case. One study showed that in some cases pulmonary function was not significantly impaired with mild pulmonary inflammation [76]. Another study revealed that small groups of soldiers who were potentially exposed to a broad range of airborne hazards (e.g., burn pits, desert dust, and sulfur dioxide) in Iraq and Afghanistan had constrictive bronchiolitis, but they had normal results on pulmonary function tests [13].

Despite these conflicting studies, our results suggest that the mixture smoke from flaming combustion is more potent and likely to be responsible for both lung toxicity and physiology effects. In support of these findings, one study indicated that hundreds of veterans who had served during the Iraq and Afghanistan wars reported lung disease, neurological disorders, and cancers in connection to burn pit smoke exposure [11]. Because burn pit smoke is a potential source of dioxin and dioxin-like compounds, exposures to such smoke is also of particular concern to military communities [29, 31, 33]. The unique environmental conditions in Iraq and Afghanistan may have contributed to greater diagnoses of asthma and respiratory symptoms among military personnel who deployed to these areas compared with those who did not deploy [5, 6, 20].

### Mutagenicity of the burn pit smoke PM associated with PAHs

PAHs are a group of chemicals that are produced mainly by incomplete combustion of organic materials, and many of them are classified as carcinogenic and mutagenic to humans [55, 56, 77]. Notably, their mutagenic effects are more pronounced when they have nitro-functional groups (nitro-PAHs) compared to parent PAHs [78]. We demonstrated that on an equal mass basis, the highest flaming mutagenic potency of any sample in TA100 + S9 was the mixture/diesel at 9.308 rev/µg PM (Table 2). This sample also had the highest total sum of PAHs of any sample (68,901 µg PAHs/g PM, Additional file 1: Table S2). In contrast, the flaming sample with the lowest mutagenic potency in TA100 + S9 was the cardboard (2.497 rev/µg PM, Table 2); it also had the lowest total sum of PAHs of any sample (8,582 µg PAHs/g PM, Additional file 1: Table S2). We observed a strong correlation between the mutagenic potency of the PM in TA100 + S9, which detects primarily PAHs, and the total sum of PAHs per g PM for all the samples (Pearson’s *r* = 0.97, Additional file 1: Fig. S4).

The higher mutagenic potency of the flaming smoke samples was consistent with findings from our previous study of biomass smoke [49]. This is likely because the temperatures of combustion were not high enough during the smoldering phase to produce PAHs [56]. Consistent with the lack of mutagenicity of the smoldering smoke samples in all three strains, the concentrations of PAHs, nitro- and oxy-PAHs were generally lower than those of the flaming smoke samples. Overall, our results clearly showed that inferences from the various strains of *Salmonella* of the main chemical classes responsible for the mutagenicity of the burn pit smoke PM were consistent with the chemical analyses across several PAH groups.

In addition to the mutagenic potency expressed as mutagenicity per mass of PM, we presented mutagenicity EFs to reflect mutagenicity based on an equal mass of waste consumed or thermal energy of waste combustion. We compared the mutagenicity EFs of the burn pit smoke PM in TA98 + S9 to those of other combustion emissions published in the literature (Fig. 7). Among the burn pit smoke PM samples, the mutagenicity EF of the flaming plastic (approximately 15 × 10^5^ rev/MJ_th_) was similar to that of the previously reported open burning of tires (approximately 20 × 10^5^ rev/MJ_th_). The tire fire study did not distinguish between flaming and smoldering phases of combustion, and its mutagenicity EF reflects a combination of both phases [79]. Moreover, in studies of polyethylene plastic in which we also did not distinguish between the flaming and smoldering phases of combustion, we found that polyethylene plastic sheeting burned to simulate open burning had a mutagenicity EF of approximately 1.0 × 10^5^ rev/MJ_th_ [42], and polyethylene pipe burned in a rotary kiln had a mutagenicity EF of approximately 0.1 × 10^5^ rev/MJ_th_ [80]. Again, these values reflect a combination of flaming and smoldering phases of combustion and are 1–2 orders of magnitude lower than the flaming mutagenicity EF for plastic smoke found in the present study. Given the low mutagenicity EF of the smoldering plastic smoke PM, and the high mutagenicity EF of the flaming plastic emissions, it is likely that an analysis that reflected both phases would show an intermediate mutagenicity EF, which is consistent with our earlier findings for polyethylene plastic combustion emissions [42, 80].

We also found that the mutagenicity EF of the flaming plywood smoke PM was similar to those of various types of biomass smoke PM from a three-stone cookstove or simulated wildfires, which produced mainly flaming smoke emissions. The mutagenicity EFs from waste incinerators, which range from approximately 10^2^ to 10^4^ rev/MJ_th_ [42, 80–82], are generally lower than those from our simulation of open burn pits. Nonetheless, exposures to mutagenic combustion emissions are associated with various adverse health effects, including cancer [83–87]. Despite the lack of scientific data demonstrating a direct link between burn pit exposures and cancers, the presence of large quantities of carcinogenic chemicals in military burn pits is a cause for concern [29, 31, 33, 82]. In a similar vein, our results also show that the mutagenicity EFs of burn pits combusting typical types of waste disposed of in such pits are within the range associated with other types of combustion emissions, the exposure to which is associated with a variety of health effects.

## Conclusions

The burn pit smoke PM condensates emitted from a lab-scale furnace with a combustion control system induced significant increases in lung toxicity and mutagenicity. The greatest effects on an equal mass basis were observed from flaming combustion emissions of plastic or plastic containing wastes, which generated black smoke plumes and were associated with higher PAH concentrations. Despite the fact that the nature and composition of military burn pits varied widely by location and over time during the wars in Iraq and Afghanistan, our results support the notion that open burning of plastic waste is a key risk factor for adverse health outcomes. It should be noted that the bioassays presented here were limited to exposures of freshly emitted smoke condensates. However, actual exposures to military burn pits are more complicated, involving gas phase constituents, photochemical aging, burning of chlorinated waste, and repeated exposures, resulting in potentially greater and more diverse health problems. As we have shown previously, open burning, regardless of what is burned, produced higher mutagenicity EFs than controlled burning.

Although there are limited studies on the health effects of burn pit smoke exposures, our findings provide a potential cause-and-effect relationship between exposure to emissions from simulated burn pits and subsequent health outcomes. Moreover, these results could also be relevant to smoke exposure from burning homes, structures, and discarded solid materials in the wildland-urban interface (WUI) areas during wildfires. More studies are needed to investigate the impacts of acute and chronic inhalation of whole burn pit smoke, which includes gas phase components. In addition, these effects should be examined in models of disease to determine whether exposures can exacerbate pre-existing health conditions, such as asthma and cardiovascular disease. Overall, our results may lead to an improved understanding of the characteristics of these complex emissions and their impact on the health of military personnel deployed in war zones or in other circumstances where open burning of waste occurs.

## Methods

### Burn pit materials

We burned five different burn pit materials: plywood (ActionPak Inc., Bristol, PA), cardboard (ActionPak, Inc., Bristol, PA), plastic [a mixture of low-density polyethylene (LDPE), high-density polyethylene (HDPE), polyethylene terephthalate (PET), and polystyrene (PS) pellets], mixture (a mixture of plywood, plastic, and cardboard materials), and mixture/diesel (the mixture material treated with diesel). Plywood was used to represent ammunition boxes that are made of pine wood and graded to a military specification. Cardboard was used to represent cardboard papers that are made of weather-resistant corrugated papers and designed for military packing specifications. Plastic was used to represent plastic materials that are the four most prevalent types of plastic waste (PET (40 wt%), HDPE (24 wt%), LDPE (19 wt%), and PS (17 wt%) based on analysis of the solid waste stream data collected from U.S. military bases [28, 50]). Mixture was used to represent key components of military burn pits that consisted mostly of paper (49 wt%), plastic (27 wt%) and wood (24 wt%). The mass fraction of each waste material based on the waste stream data from U.S. military bases [28, 50] is summarized in Additional file 1: Table S1. Mixture/diesel (the mixture treated with 10 wt% diesel) was used to simulate a typical burn pit condition where military waste was burned with jet fuel, diesel, or gasoline to accelerate the burning at the pits [4, 5]. Diesel fuel (DF-2) was obtained from the Air Methods and Characterization Division at the U.S. EPA. All the burn pit materials were cut into approximately 1-cm long pieces to facilitate uniform combustion conditions and stored in a temperature- and humidity-controlled room (23 °C and 39% relative humidity) until used.

### Generation of burn pit smoke

We used an automated furnace system to simulate burn pit combustions under smoldering and flaming phases. The burn pit material (15 g) was placed inside the quartz tube and burned under controlled combustion phases (smoldering and flaming) for 60 min. Half of the outlet burn pit smoke flow from the furnace was captured by a multi-stage cryotrap system consisting of three sequential impingers maintained at − 10 °C, − 50 °C, and − 70 °C. The cryo-trapped smoke samples were then used for chemical analyses and toxicity tests. A detailed description of the furnace and cryotrap system is provided in our previous paper [49].

The other half of the burn pit smoke flow was monitored continuously for carbon dioxide (CO_2_) and carbon monoxide (CO) using a non-dispersive infrared analyzer (Model: 602 CO/CO_2_; CAI Inc., Orange, CA) and nitrogen oxide (NO, NO_2_, and NOx) using a chemiluminescent analyzer (Model: 42i NO/NO_2_/NOx; Thermo Scientific, Franklin, MA). Volatile organic compounds (VOCs) were also sampled from the burn pit smoke for further speciation and analysis. VOCs in the smoke were sampled using 6-L SUMMA canisters, and carbonyls were sampled with 2,4-dinitrophenylhydrazine (DNPH)-coated silica cartridges (PN 505323, Sigma-Aldrich Co., St. Louis, MO).

The sampling flow rates through the evacuated canister (filled to approximately 0.7 atm) were controlled using a critical orifice at a flow rate of approximately 70 mL/min. Cartridge sampling flow rates were controlled with a SKC Aircheck Sampling Pump (Model: 224-PCXR8, SKC Inc., Eighty Four, PA) with flow rates in the range of 0.5–0.7 L/min.

PM was collected on a glass-fiber filter to determine mean PM concentrations gravimetrically by weighing the filter before and after combustion. Particle-size distributions (in the range of 10 nm to 10 µm) were monitored using a scanning mobility particle sizer (NanoScan SMPS, Model:3910; TSI Inc., Shoreview, MN) combined with an optical particle sizer (OPS, Model: 3330; TSI Inc.).

### Characterization of burn pit smoke

CO_2_, CO, and PM concentrations were used to characterize the burn pit smoke emissions. Flaming and smoldering combustion phases are typically characterized by modified combustion efficiency (MCE), which is defined as MCE = ∆CO_2_/(∆CO_2_ + ∆CO), where ∆CO_2_ and ∆CO are the excess concentrations of CO_2_ and CO [88]. In this study, combustion was considered flaming when the MCE was > 0.95 and smoldering when the MCE was 0.65–0.85 [89].

Smoke properties are also described using emission factors (EFs), which are defined as the mass of species *t* emitted per mass of dry fuel consumed, which can be calculated as EF *t* (g/kg) = (F*c* × C*t* × M*t* × 1000)/(M*c* × C_*T*_), where F*c* is the mass fraction of carbon in the dry burn pit material [28], M*t* is the molar mass of species *t*, M*c* is the molar mass of carbon, C_*T*_ is the total mass of carbon contributed by all species in the burn pit smoke, and C*t* is the mass of carbon emitted as species *t*, and given by C*t* (mg/m^3^) = (M*c* × N × V*t*)/24.45, where N is the number of carbon atoms in species *t*, and V*t* is the concentration of species *t* in ppm [90]. In order to validate EFs estimated from the furnace in the present study, EFs for CO, CO_2_, and PM were compared with the published EFs from various biomass combustion conditions.

### Chemical analysis of burn pit smoke

VOCs in the canisters were analyzed by gas chromatography-mass spectrometry (GC–MS) in accordance with U.S. EPA Method TO-15. DNPH cartridge samples were extracted with 6 mL of carbonyl-free acetonitrile (Burdick & Jackson, VWR International, Radnor, PA). Carbonyl hydrazones were analyzed in the extracts by high performance liquid chromatography (HPLC) according to U.S. EPA Method TO-11A. Detailed descriptions of the TO-15 and TO-11A analytical procedures were reported previously [91]. Note that the VOC measurement methods used in this study target the most important air toxics and ozone precursors and does not represent a complete VOC speciation in burn pit smoke emissions.

PM was extracted from the cryogenically cooled impingers by washing with acetone. For carbon species analysis, an aliquot of the smoke PM suspension was pipetted onto pre-baked 1.5-cm^2^ quartz filter punches, dried, and analyzed for organic carbon (OC) and elemental carbon (EC) with a thermal-optical analyzer (Model: 107A; Sunset Laboratory Inc., Tigard, OR). The OC fraction was further analyzed for PAHs. Sixteen PAHs as priority pollutants classified by the U.S. Environmental Protection Agency (EPA) and 16 nitro-/oxy-PAHs were analyzed by GC–MS (Model: 7890/5975B GC/MSD system, Agilent Technologies Inc., Santa Clara, CA). PAHs were chromatographed using a capillary column (30 m × 0.25 mm × 0.25 µm; BD-5) ramped from 60 to 300 °C with a pulsed splitless injection of 2 µl of PM suspension. Quantification of PAHs was based on the isotope dilution method. Detection limits were established for each target listing. Raw values that fell below the detection limit threshold were listed as not detected (ND).

### Experimental animals

Adult pathogen-free female CD-1 mice (approximately 28 g body weight) were purchased from Charles River Breeding Laboratories (Raleigh, NC), housed in groups of 3 in polycarbonate cages with hardwood chip bedding at the U.S. EPA Animal Care Facility (accredited by the Association for Assessment and Accreditation of Laboratory Animal Care), and were maintained on a 12-h light-to-dark cycle at 22.3 ± 1.1 °C temperature and 50 ± 10% relative humidity. Mice were weighed and weight-randomized into 24 groups of 6 mice each for each exposure condition. Mice were given access to rodent chow and water ad libitum and were acclimated for at least 10 days before the study began. Mice were treated humanely and with regard for alleviation of suffering. This study was conducted after approval by the EPA Institutional Animal Care and Use Committee.

### Exposure to burn pit smoke PM

Use of the cryotrap sampling technique is unique in that it collects semivolatile organic compounds in all physical states. Thus, the PM condensate used for toxicological assessments contains the total individual semivolatile organic compounds concentrations within the whole smoke mixture, which is pertinent for exposure. We solvent-exchanged the smoke PM suspension in acetone into saline to a final PM concentration of 2 mg/mL and then administered it into the lungs of CD-1 mice at 100 µg in 50 µL by oropharyngeal aspiration. We performed oropharyngeal aspiration on mice anesthetized in a small plexiglass box using vaporized anesthetic isoflurane as described previously [49]. The selection of PM dose (100 µg) was based on extreme exposure levels of PM (> 1000 µg/m^3^) at military bases in Iraq, where the largest burn pit was operated [6, 64]. If exposures are near or close to the burn pits (assuming > 3000 µg/m^3^ of PM), PM deposited in the human lungs for 24 h would be 123 ng/cm^2^ [49]. In the present study, the PM dose (100 µg) to the mouse lung was calculated to be 126 ng/cm^2^, assuming the respiratory deposition fraction by the oropharyngeal aspiration method and surface area of 0.81 and 642 cm^2^, respectively [92, 93], and appeared to be relevant to the inhaled burn pit smoke PM concentrations in human lungs (123 ng/cm^2^). We instilled additional mice with 2 µg of lipopolysaccharide in 50 µL saline (LPS; *Escherichia coli* endotoxin; 0111:B4 containing 10^6^ unit/mg material, Sigma, St. Louis, MO) as a positive control to demonstrate maximal responsiveness to this well characterized inflammatory agent. We also instilled additional mice with 50 µL saline alone as a negative control.

### Breathing parameter assessment

Mice were tested for breathing parameters before exposures, and after exposure approximately 1 h before euthanasia. Breathing parameters were assessed using a whole-body plethysmography (WBP) system (Emka Technologies, Falls Church, VA) as previously described [94]. Breathing parameters measured include minute ventilation (MV), tidal volume (TV), breathing frequency (F), relaxation time (RT), inspiratory (Ti) and expiratory (Te) time, and peak inspiratory (PIF) and peak expiratory (PEF) flow. In this system the mouse had complete freedom of movement in a small clear plastic chamber (3.5" diameter × 2.5" height).

### Lung toxicity assay

At 4- and 24-h post-exposure, mice were euthanized by overdose with sodium pentobarbital and phenytoin sodium i.p. (Euthasol; Virbac AH Inc., Fort Worth, TX). Blood was collected by cardiac puncture, and hematology values were measured using a Coulter AcT 10 Hematology Analyzer (Beckman Coulter Inc., Miami, FL). Bronchoalveolar lavage fluid (BALF) was collected from the right lung lobes and used to determine the total cell count and differential analysis of macrophage and neutrophil numbers. Total BALF cell count of each mouse was obtained by a Coulter counter (Coulter Co., Miami, FL). Concentrations of lactate dehydrogenase (LDH) in BALF were determined using commercially available kits (LDH-L Reagent, Thermo Scientific, Middletown, VA). This assay was modified for use on the KONELAB 30 clinical chemistry spectrophotometer analyzer (Thermo Clinical Lab Systems, Espoo, Finland) as described previously [49]. Concentrations of tumor necrosis factor-α (TNF-α), interleukin-6 (IL-6), and macrophage inhibitory protein-2 (MIP-2) in BALF were determined using commercial multiplexed fluorescent bead-based immunoassays (Milliplex Map Kit, Millipore Co., Billerica, MA) measured by a Luminex 100 (Luminex Co., Austin, TX) following the manufacturer’s protocol. The limits of detection (LOD) of each cytokine were 6.27, 3.28, and 29.14 pg/mL for TNF-α, IL-6, and MIP-2, respectively. All values below these lowest values were replaced with a fixed value of one-half of the LOD value.

### Mutagenicity assay

For mutagenicity analysis, we extracted the organics from the smoke PM with dichloromethane (DCM), determined the percentage of extractable organic material (%EOM) by gravimetric analysis, and then solvent-exchanged the EOM into dimethyl sulfoxide (DMSO) as described [49]. We performed the *Salmonella* plate-incorporation mutagenicity assay [95] using the base-substitution strain TA100 [*hisG46 chl-1005* (*bio uvrB gal*) *rfa-1001* pKM101 + Fels-1 + Fels-2 + Gifsy-1 + Gifsy-2 +] and the frameshift strain TA98 [*hisD3052 chl-1008* (*bio uvrB gal*) *rfa-1001* pKM101 + Fels-1 + Fels-2 + Gifsy-1 + Gifsy-2 +] [96].

The *Salmonella* (Ames) mutagenicity assay is the standard assay, now in use for 50 years, for evaluating the mutagenicity of complex mixtures [97]. As indicated below, the various strains have metabolic and genetic factors that permit them to detect preferentially certain chemical classes of mutagens, such as PAHs, nitro-PAHs, or aromatic amines [97]. For example, strain TA98 is especially sensitive to nitro-PAHs (nitroarenes) because it detects mutagens that induce frameshift mutations, which is the main class of mutation induced by most nitro-PAHs. In addition, in the absence of any exogenous metabolic activation (rat liver S9), TA98 contains enough nitroreductase to activate nitro-PAHs to frameshift mutagens. In contrast, in the presence of S9, this strain detects aromatic amines because the S9 can acetylate aromatic amines or activated nitro-PAHs into acetylated aromatic amines, which can bind to DNA (typically guanine), forming DNA adducts; aromatic amines preferentially induce frameshifts. This strain, as well as TA100, are both missing nucleotide excision repair and, thus, they cannot repair such DNA adducts. Finally, these adducts are processed by the error-prone DNA polymerase (pKM101) that is present in both TA98 and TA100, which makes a mutation opposite these unrepaired DNA adducts. TA100 detects mutagens such as PAHs, which induce primarily base substitutions in the presence of S9, resulting in epoxides, which bind to DNA (typically guanine), and as noted above, such adducts cannot be repaired in this strain, and instead, the pKM101 polymerase processes this into a base-substitution mutation. Given sample limitation, we did not evaluate the samples in strain TA100 -S9, which would detect direct-acting base-substitution mutagens of various chemical classes. We evaluated the EOM in the presence and absence of metabolic activation using S9 mix composed of 1 mg S9 protein/500 µL of S9 mix [95]. The S9 was an Aroclor-induced Sprague–Dawley rat liver homogenate purchased from Moltox (Boone, NC). 2-Aminoanthracene (0.5μg/plate) for TA98 + S9 and TA100 + S9, and 2-nitrofluorene (3μg/plate) for TA98 -S9 (both from Sigma, St. Louis, MO) were both dissolved in DMSO and used as positive controls, and DMSO was used as a negative control.

The burn pit smoke samples were evaluated among 9 doses (2.5, 5, 10, 25, 50, 100, 250, 500, and 1,000 µg EOM/plate) at one plate/dose. The smoldering samples were evaluated in 4 independent experiments, and the flaming samples were evaluated in 2 independent experiments; limited sample prevented a third experiment with the flaming samples. We defined a positive mutagenic response as a reproducible, dose-related increase in revertants (rev) per plate relative to the DMSO control. The mutagenic potencies of the EOM (rev/µg EOM) were the slopes of the linear regressions over the initial linear portion of the dose–response curves created by combining the data (rev/plate) from the independent experiments. Samples were considered mutagenic when analysis of the curves by a trend test showed significance at *P* < 0.05. We multiplied the mutagenic potencies of the EOM (rev/µg EOM) by the %EOM to give the mutagenic potencies of the PM (rev/µg PM) for each burn pit smoke.

To calculate the mutagenicity emission factors (EFs), we multiplied the rev/µg PM by 10^6^ to give rev/kg PM, and these values were then multiplied by the g PM/kg fuel that we determined experimentally to give the mutagenicity emission factor (EF) expressed as rev/kg fuel. We converted rev/kg fuel to rev/MJ_th_ by dividing the rev/kg fuel values by the heat energy of the burn pit (MJ_th_/kg) based on the following values: 20.0 for plywood [98], 17.5 for cardboard [99], 32.5 for plastic [100], and 22.6 for the mixture and the mixture/diesel [100]. We compared the results to rev/MJ_th_ values determined for a variety of other combustion emissions available from the literature.

### Statistical analysis

For the analysis of mutagenicity data (TA98 ± S9 and TA100 + S9) and lung toxicity data (pro-inflammatory cytokines, LDH, and hematology values), we used one-way analysis of variance (ANOVA) followed by Tukey multiple comparison or the Fisher’s Least Significant Difference test to compare the biological responses. For the analysis of lung function data, we used two-way analysis of variance (ANOVA) followed by Sidak multiple comparison to compare the time course of the respiratory parameters between the smoke-exposed groups and the saline control group. This analysis was performed using GraphPad Prism software (version 6.07, GraphPad Software Inc., San Diego, CA). We also modeled the lung toxicity potencies (# neutrophils) of the burn pit smoke PM with negative binomial regression in R Statistical Software (version 3.3.2, R Foundation for Statistical Computing, Vienna, Austria) [49]. Pearson correlation analyses and linear regressions were used to analyze relationship between toxicity outcomes and total PAH concentrations of the burn pit smoke PM and performed with Microsoft excel (Microsoft Co., Redmond, WA). Data were expressed as mean ± standard error of the mean (SEM). The statistical significance level was assigned at a probability value of *P* < 0.05.

## Supplementary Information

**Additional file 1: Table S1.** Military Waste Stream Analysis (% by Weight). **Table S2.** Concentration (µg/g) of PAHs in the burn pit smoke PM. **Table S3.** Breathing parameters from the burn pit smoke exposures. **Figure S1.** Emission factors for VOCs of the burn pit smoke emissions. **Figure S2.** Mutagenicity dose–response curves of the smoldering samples in *Salmonella* strain TA98 with or without metabolic activation (S9). **Figure S3.** Mutagenicity dose–response curves of the flaming samples in *Salmonella* strains TA98 and TA100 with or without metabolic activation (S9). **Figure S4.** Correlation between biological responses and total PAH concentration of PM.

## Data Availability

Datasets associated with this study are available from the corresponding authors.

## Abbreviations

PM: Particulate matter
PAH: Polycyclic aromatic hydrocarbon
WHO: World Health Organization
WUI: Wildland-urban interface
VOC: Volatile organic compound
EF: Emission factor
BALF: Bronchoalveolar lavage fluid
LPS: Lipopolysaccharide
LDH: Lactate dehydrogenase
IL-6: Interleukin-6
MIP-2: Macrophage inflammatory protein-2
TNF-α: Tumor necrosis factor-α
MV: Minute ventilation
TV: Tidal volume
F: Breathing frequency
RT: Relaxation time
Ti: Inspiratory time
Te: Expiratory time
PIF: Peak inspiratory flow
PEF: Peak expiratory flow
EOM: Extractable organic material
LDPE: Low density polyethylene
HDPE: High density polyethylene
PET: Polyethylene terephthalate
PS: Polystyrene
DNPH: 2,4-Dinitrophenylhydrazine
MCE: Modified combustion efficiency
GC–MS: Gas chromatography-mass spectrometry
HPLC: High performance liquid chromatography
OC: Organic carbon
EC: Elemental carbon
WBP: Whole-body plethysmography
DCM: Dichloromethane
DMSO: Dimethyl sulfoxide
Rev: Revertants
ANOVA: Analysis of variance
SEM: Standard error of the mean

## References

[1] WHO (2017). Preventing noncommunicable diseases (NCDs) by reducing environmental risk factors.

[2] Keita S, Liousse C, Yoboué V, Dominutti P, Guinot B, Assamoi EM (2018). Particle and VOC emission factor measurements for anthropogenic sources in West Africa. Atmos Chem Phys.

[3] Lemieux PM, Lutes CC, Santoianni DA (2004). Emissions of organic air toxics from open burning: a comprehensive review. Prog Energy Combust Sci.

[4] IOM (2011). Long-term health consequences of exposure to burn pits in Iraq and Afghanistan.

[5] Szema A, Mirsaidi N, Patel B, Viens L, Forsyth E, Li J (2017). Proposed Iraq/Afghanistan war-lung injury (IAW-LI) clinical practice recommendations: National Academy of Sciences' Institute of Medicine Burn Pits Workshop. Am J Mens Health.

[6] Szema AM, Peters MC, Weissinger KM, Gagliano CA, Chen JJ (2010). New-onset asthma among soldiers serving in Iraq and Afghanistan. Allergy Asthma Proc.

[7] Butler DA, Styka AN, Savitz DA (2017). Assessment of the Department of Veterans Affairs Airborne Hazards and Open Burn Pit Registry.

[8] DoD. Open Burn Pit Report to Congress. In: Defense Do, editor. Secretary of defense for acquisition and sustainment; 2019. p. 1–7.

[9] Baiocchi D (2013). Measuring army deployments to Iraq and Afghanistan.

[10] Coughlin SS, Szema A (2019). Burn pits exposure and chronic respiratory illnesses among Iraq and Afghanistan veterans. J Environ Health Sci.

[11] Furlow B (2010). US Institute of Medicine studies military burn pits. Lancet Oncol.

[12] Helmer DA, Rossignol M, Blatt M, Agarwal R, Teichman R, Lange G (2007). Health and exposure concerns of veterans deployed to Iraq and Afghanistan. J Occup Environ Med.

[13] King MS, Eisenberg R, Newman JH, Tolle JJ, Harrell FE, Nian H (2011). Constrictive bronchiolitis in soldiers returning from Iraq and Afghanistan. N Engl J Med.

[14] Liu J, Lezama N, Gasper J, Kawata J, Morley S, Helmer D (2016). Burn pit emissions exposure and respiratory and cardiovascular conditions among airborne hazards and open burn pit registry participants. J Occup Environ Med.

[15] Mallon CT, Rohrbeck MP, Haines MK, Jones DP, Utell M, Hopke PK (2016). Introduction to Department of Defense research on burn pits, biomarkers, and health outcomes related to deployment in Iraq and Afghanistan. J Occup Environ Med.

[16] Sharkey JM, Abraham JH, Clark LL, Rohrbeck P, Ludwig SL, Hu Z (2016). Postdeployment respiratory health care encounters following deployment to Kabul, Afghanistan: a retrospective cohort study. Mil Med.

[17] Szema AM (2013). Occupational lung diseases among soldiers deployed to Iraq and Afghanistan. Occup Med Health Aff.

[18] Woeller CF, Thatcher TH, Van Twisk D, Pollock SJ, Croasdell A, Hopke PK (2016). MicroRNAs as novel biomarkers of deployment status and exposure to polychlorinated dibenzo-p-dioxins/dibenzofurans. J Occup Environ Med.

[19] Shorr AF, Scoville SL, Cersovsky SB, Shanks GD, Ockenhouse CF, Smoak BL (2004). Acute eosinophilic pneumonia among US Military personnel deployed in or near Iraq. JAMA.

[20] Smith B, Wong CA, Smith TC, Boyko EJ, Gackstetter GD (2009). Newly reported respiratory symptoms and conditions among military personnel deployed to Iraq and Afghanistan: a prospective population-based study. Am J Epidemiol.

[21] Engelbrecht JP, McDonald EV, Gillies JA, Jayanty RK, Casuccio G, Gertler AW (2009). Characterizing mineral dusts and other aerosols from the Middle East-part 1: ambient sampling. Inhal Toxicol.

[22] Abraham JH, Eick-Cost A, Clark LL, Hu Z, Baird CP, DeFraites R (2014). A retrospective cohort study of military deployment and postdeployment medical encounters for respiratory conditions. Mil Med.

[23] Conlin AM, DeScisciolo C, Sevick CJ, Bukowinski AT, Phillips CJ, Smith TC (2012). Birth outcomes among military personnel after exposure to documented open-air burn pits before and during pregnancy. J Occup Environ Med.

[24] Erdtmann F (2015). Long-term health consequences of exposure to burn pits in Iraq and Afghanistan. Mil Med.

[25] Falvo MJ, Bradley M, Brooks SM (2014). Is deployment an "exposure" in military personnel?. J Occup Environ Med.

[26] Powell TM, Smith TC, Jacobson IG, Boyko EJ, Hooper TI, Gackstetter GD (2012). Prospective assessment of chronic multisymptom illness reporting possibly associated with open-air burn pit smoke exposure in Iraq. J Occup Environ Med.

[27] Smith B, Wong CA, Boyko EJ, Phillips CJ, Gackstetter GD, Ryan MA (2012). The effects of exposure to documented open-air burn pits on respiratory health among deployers of the Millennium Cohort Study. J Occup Environ Med.

[28] Aurell J, Barnes M, Gullett BK, Holder A, Eninger R (2019). Methodology for characterizing emissions from small (0.5–2 MTD) batch-fed gasification systems using multiple waste compositions. Waste Manag.

[29] Aurell J, Gullett BK, Yamamoto D (2012). Emissions from open burning of simulated military waste from forward operating bases. Environ Sci Technol.

[30] Gullett BK, Touati A, Hays MD (2003). PCDD/F, PCB, HxCBz, PAH, and PM emission factors for fireplace and woodstove combustion in the San Francisco Bay region. Environ Sci Technol.

[31] Masiol M, Mallon CT, Haines KM, Utell MJ, Hopke PK (2016). Airborne dioxins, furans, and polycyclic aromatic hydrocarbons exposure to military personnel in Iraq. J Occup Environ Med.

[32] Verma R, Vinoda KS, Papireddy M, Gowda ANS (2016). Toxic pollutants from plastic waste: a review. Procedia Environ Sci.

[33] Weese CB (2010). Issues related to burn pits in deployed settings. US Army Med Dep J.

[34] de Souza CV, Corrêa SM (2016). Polycyclic aromatic hydrocarbons in diesel emission, diesel fuel and lubricant oil. Fuel.

[35] Fine PM, Cass GR, Simoneit BR (2001). Chemical characterization of fine particle emissions from fireplace combustion of woods grown in the Northeastern United States. Environ Sci Technol.

[36] Fine PM, Cass GR, Simoneit BR (2002). Chemical characterization of fine particle emissions from the fireplace combustion of woods grown in the Southern United States. Environ Sci Technol.

[37] Hays MD, Gullett B, King C, Robinson J, Preston W, Touati A (2011). Characterization of carbonaceous aerosols emitted from outdoor wood boilers. Energy Fuels.

[38] Hays MD, Kinsey J, George I, Preston W, Singer C, Patel B (2019). Carbonaceous particulate matter emitted from a pellet-fired biomass boiler. Atmosphere.

[39] Hays MD, Smith ND, Kinsey J, Dong Y, Kariher P (2003). Polycyclic aromatic hydrocarbon size distributions in aerosols from appliances of residential wood combustion as determined by direct thermal desorption—GC/MS. J Aerosol Sci.

[40] Hedberg E, Kristensson A, Ohlsson M, Johansson C, Johansson P-Å, Swietlicki E (2002). Chemical and physical characterization of emissions from birch wood combustion in a wood stove. Atmos Environ.

[41] Kleindienst TE, Shepson PB, Edney EO, Claxton LD, Cupitt LT (1986). Wood smoke: measurement of the mutagenic activities of its gas- and particulate-phase photooxidation products. Environ Sci Technol.

[42] Linak WP, Ryan JV, Perry E, Williams RW, DeMarini DM (1989). Chemical and biological characterization of products of incomplete combustion from the simulated field burning of agricultural plastic. JAPCA.

[43] McDonald JD, Zielinska B, Fujita EM, Sagebiel JC, Chow JC, Watson JG (2000). Fine particle and gaseous emission rates from residential wood combustion. Environ Sci Technol.

[44] McMeeking GR, Kreidenweis SM, Baker S, Carrico CM, Chow JC, Collett JL (2009). Emissions of trace gases and aerosols during the open combustion of biomass in the laboratory. J Geophys Res Atmos.

[45] Mutlu E, Warren SH, Ebersviller SM, Kooter IM, Schmid JE, Dye JA (2016). Mutagenicity and pollutant emission factors of solid-fuel cookstoves: comparison with other combustion sources. Environ Health Perspect.

[46] Mutlu E, Warren SH, Matthews PP, Schmid JE, Kooter IM, Linak WP (2015). Health effects of soy-biodiesel emissions: bioassay-directed fractionation for mutagenicity. Inhal Toxicol.

[47] Orasche J, Seidel T, Hartmann H, Schnelle-Kreis J, Chow JC, Ruppert H (2012). Comparison of emissions from wood combustion. Part 1: emission factors and characteristics from different small-scale residential heating appliances considering particulate matter and polycyclic aromatic hydrocarbon (PAH)-related toxicological potential of particle-bound organic species. Energy Fuels.

[48] Schauer JJ, Kleeman MJ, Cass GR, Simoneit BRT (2001). Measurement of emissions from air pollution sources. 3. C1–C29 organic compounds from fireplace combustion of wood. Environ Sci Technol.

[49] Kim YH, Warren SH, Krantz QT, King C, Jaskot R, Preston WT (2018). Mutagenicity and lung toxicity of smoldering vs. flaming emissions from various biomass fuels: implications for health effects from wildland fires. Environ Health Perspect.

[50] USALIA. US Army Central (USARCENT) area of responsibility (AOR) contigency base waste stream analysis. 2013.

[51] Kohse-Höinghaus K (2020). Combustion in the future: the importance of chemistry. Proc Combust Inst.

[52] Hoffer A, Jancsek-Turóczi B, Tóth Á, Kiss G, Naghiu A, Levei EA (2020). Emission factors for PM10 and polycyclic aromatic hydrocarbons (PAHs) from illegal burning of different types of municipal waste in households. Atmos Chem Phys.

[53] Bolling AK, Totlandsdal AI, Sallsten G, Braun A, Westerholm R, Bergvall C (2012). Wood smoke particles from different combustion phases induce similar pro-inflammatory effects in a co-culture of monocyte and pneumocyte cell lines. Part Fibre Toxicol.

[54] Kim KS, Hong KH, Ko YH, Kim MG (2004). Emission characteristics of PCDD/Fs, PCBs, chlorobenzenes, chlorophenols, and PAHs from polyvinylchloride combustion at various temperatures. J Air Waste Manag Assoc.

[55] Abdel-Shafy HI, Mansour MSM (2016). A review on polycyclic aromatic hydrocarbons: source, environmental impact, effect on human health and remediation. Egypt J Pet.

[56] IARC. Some non-heterocyclic polycyclic aromatic hydrocarbons and some related exposures. IARC monographs on the evaluation of carcinogenic risks to humans. No. 92. 2010/12/15 ed. Lyon, France: International Agency for Research on Cancer (IARC) Working Group on the Evaluation of Carcinogenic Risks to Humans; 2010. p. 1–853. PMC4781319 21141735

[57] Katari VS, Vatavuk WM, Wehe AH (1987). Incineration techniques for control of volatile organic compound emissions. Part I: fundamentals and process design considerations. JAPCA.

[58] Sekimoto K, Koss AR, Gilman JB, Selimovic V, Coggon MM, Zarzana KJ (2018). High- and low-temperature pyrolysis profiles describe volatile organic compound emissions from western US wildfire fuels. Atmos Chem Phys.

[59] Banton MI, Bus JS, Collins JJ, Delzell E, Gelbke HP, Kester JE (2019). Evaluation of potential health effects associated with occupational and environmental exposure to styrene: an update. J Toxicol Environ Health B Crit Rev.

[60] Snyder R (2000). Overview of the toxicology of benzene. J Toxicol Environ Health A.

[61] Werder EJ, Engel LS, Richardson DB, Emch ME, Gerr FE, Kwok RK (2018). Environmental styrene exposure and neurologic symptoms in U.S. Gulf coast residents. Environ Int.

[62] IARC. Chemical agents and related occupations. IARC monographs on the evaluation of carcinogenic risks to humans. No. 100F. Lyon, France: International Agency for Research on Cancer (IARC) Working Group on the Evaluation of Carcinogenic Risks to Humans; 2012. PMC4781612 23189753

[63] IARC. Styrene, styrene-7,8-oxide, and quinoline. IARC monographs on the evaluation of carcinogenic risks to humans. No. 121. Lyon, France: International Agency for Research on Cancer (IARC) Working Group on the Evaluation of Carcinogenic Risks to Humans; 2019. 31967769

[64] Falvo MJ, Osinubi OY, Sotolongo AM, Helmer DA (2015). Airborne hazards exposure and respiratory health of Iraq and Afghanistan veterans. Epidemiol Rev.

[65] Holme JA, Brinchmann BC, Refsnes M, Låg M, Øvrevik J (2019). Potential role of polycyclic aromatic hydrocarbons as mediators of cardiovascular effects from combustion particles. Environ Health.

[66] Vogel CFA, Van Winkle LS, Esser C, Haarmann-Stemmann T (2020). The aryl hydrocarbon receptor as a target of environmental stressors: implications for pollution mediated stress and inflammatory responses. Redox Biol.

[67] Marris CR, Kompella SN, Miller MR, Incardona JP, Brette F, Hancox JC (2020). Polyaromatic hydrocarbons in pollution: a heart-breaking matter. J Physiol.

[68] Peterson BS, Rauh VA, Bansal R, Hao X, Toth Z, Nati G (2015). Effects of prenatal exposure to air pollutants (polycyclic aromatic hydrocarbons) on the development of brain white matter, cognition, and behavior in later childhood. JAMA Psychiatry.

[69] Rager JE, Clark J, Eaves LA, Avula V, Niehoff NM, Kim YH (2021). Mixtures modeling identifies chemical inducers versus repressors of toxicity associated with wildfire smoke. Sci Total Environ.

[70] Rijneveld AW, van den Dobbelsteen GP, Florquin S, Standiford TJ, Speelman P, van Alphen L (2002). Roles of interleukin-6 and macrophage inflammatory protein-2 in pneumolysin-induced lung inflammation in mice. J Infect Dis.

[71] Huggett C, Levin BC (1987). Toxicity of the pyrolysis and combustion products of poly(vinyl chlorides): a literature assessment. Fire Mater.

[72] Riedel TP, DeMarini DM, Zavala J, Warren SH, Corse EW, Offenberg JH (2018). Mutagenic atmospheres resulting from the photooxidation of aromatic hydrocarbon and NOx mixtures. Atmos Environ.

[73] Zavala J, Krug JD, Warren SH, Krantz QT, King C, McKee J (2018). Evaluation of an air quality health index for predicting the mutagenicity of simulated atmospheres. Environ Sci Technol.

[74] Baraldo S, Turato G, Saetta M (2012). Pathophysiology of the small airways in chronic obstructive pulmonary disease. Respiration.

[75] Mailhot-Larouche S, Deschênes L, Lortie K, Gazzola M, Marsolais D, Brunet D (2018). Assessment of respiratory function in conscious mice by double-chamber plethysmography. J Vis Exp.

[76] Martinez ME, Harder OE, Rosas LE, Joseph L, Davis IC, Niewiesk S (2020). Pulmonary function analysis in cotton rats after respiratory syncytial virus infection. PLoS ONE.

[77] Yu H (2002). Environmental carcinogenic polycyclic aromatic hydrocarbons: photochemistry and phototoxicity. J Environ Sci Health C Environ Carcinog Ecotoxicol Rev.

[78] Bandowe BAM, Meusel H (2017). Nitrated polycyclic aromatic hydrocarbons (nitro-PAHs) in the environment: a review. Sci Total Environ.

[79] DeMarini DM, Lemieux PM, Ryan JV, Brooks LR, Williams RW (1994). Mutagenicity and chemical analysis of emissions from the open burning of scrap rubber tires. Environ Sci Technol.

[80] DeMarini DM, Williams RW, Perry E, Lemieux PM, Linak WP (1992). Bioassay-directed chemical analysis of organic extracts of emissions from a laboratory-scale incinerator: combustion of surrogate compounds. Combust Sci Technol.

[81] Linak WP, Mulholland JA, McSorley JA, Hall RE, Srivastava RK, Ryan JV (1991). Application of staged combustion and reburning to the co-firing of nitrogen-containing wastes. Hazard Waste Hazard Mater.

[82] Watts RR, Lemieux PM, Grote RA, Lowans RW, Williams RW, Brooks LR (1992). Development of source testing, analytical, and mutagenicity bioassay procedures for evaluating emissions from municipal and hospital waste combustors. Environ Health Perspect.

[83] Adetona O, Ozoh OB, Oluseyi T, Uzoegwu Q, Odei J, Lucas M (2020). An exploratory evaluation of the potential pulmonary, neurological and other health effects of chronic exposure to emissions from municipal solid waste fires at a large dumpsite in Olusosun, Lagos, Nigeria. Environ Sci Pollut Res Int.

[84] IARC. Household use of solid fuels and high-temperature frying. IARC monographs on the evaluation of carcinogenic risks to humans. No. 95. Lyon, France: International Agency for Research on Cancer (IARC) Working Group on the Evaluation of Carcinogenic Risks to Humans; 2010. PMC5046077 20701241

[85] McLean J, Anderson D, Capra G, Riley CA (2021). The potential effects of burn pit exposure on the respiratory tract: a systematic review. Mil Med.

[86] Navarro KM, Kleinman MT, Mackay CE, Reinhardt TE, Balmes JR, Broyles GA (2019). Wildland firefighter smoke exposure and risk of lung cancer and cardiovascular disease mortality. Environ Res.

[87] Sun S, Schiller JH, Gazdar AF (2007). Lung cancer in never smokers–a different disease. Nat Rev Cancer.

[88] Ward DE, Hardy CC (1991). Smoke emissions from wildland fires. Environ Int.

[89] Urbanski S (2014). Wildland fire emissions, carbon, and climate: emission factors. For Ecol Manag.

[90] Soares Neto TG, Carvalho JA, Veras CAG, Alvarado EC, Gielow R, Lincoln EN (2009). Biomass consumption and CO_2_, CO and main hydrocarbon gas emissions in an Amazonian forest clearing fire. Atmos Environ.

[91] George IJ, Hays MD, Snow R, Faircloth J, George BJ, Long T (2014). Cold temperature and biodiesel fuel effects on speciated emissions of volatile organic compounds from diesel trucks. Environ Sci Technol.

[92] Foster WM, Walters DM, Longphre M, Macri K, Miller LM (2001). Methodology for the measurement of mucociliary function in the mouse by scintigraphy. J Appl Physiol.

[93] Weibel ER (1973). Morphological basis of alveolar-capillary gas exchange. Physiol Rev.

[94] Kim YH, King C, Krantz T, Hargrove MM, George IJ, McGee J (2019). The role of fuel type and combustion phase on the toxicity of biomass smoke following inhalation exposure in mice. Arch Toxicol.

[95] Maron DM, Ames BN (1983). Revised methods for the Salmonella mutagenicity test. Mutat Res.

[96] Porwollik S, Wong RM, Sims SH, Schaaper RM, DeMarini DM, McClelland M (2001). The DeltauvrB mutations in the Ames strains of Salmonella span 15 to 119 genes. Mutat Res.

[97] Claxton LD, Umbuzeiro Gde A, DeMarini DM (2010). The Salmonella mutagenicity assay: the stethoscope of genetic toxicology for the 21st century. Environ Health Perspect.

[98] Nielson RW, Dobie J, Wright DM. Conversion factors for the forest product industry in western Canada. Forintek Canada Corp.: Western Laboratory; 1985. Contract No.: SP-24R.

[99] Cushman-Roisin B, Cremonini B (2021). Data, statistics, and useful numbers for environmental sustainability.

[100] Themelis NJ, Castaldi MN, Bhatti J, Arsova L (2011). Energy and economic value of non-recycled plastic (NRP) and municipal solid wastes (MSW) that are currently landfilled in the fifty states.

[101] Chandrasekaran SR, Hopke PK, Hurlbut A, Newtown M (2013). Characterization of emissions from grass pellet combustion. Energy Fuels.

[102] DeMarini DM, Warren SH, Lavrich K, Flen A, Aurell J, Mitchell W (2017). Mutagenicity and oxidative damage induced by an organic extract of the particulate emissions from a simulation of the deepwater horizon surface oil burns. Environ Mol Mutagen.

