# Supplementary material for: Chemistry, lung toxicity and mutagenicity of burn pit smoke-related particulate matter
Source: Part Fibre Toxicol. 2021 Dec 16;18:45. doi: 10.1186/s12989-021-00435-w (PMC8675519; doi:10.1186/s12989-021-00435-w)
Supplement: Supplementary file 1 — Additional file 1: Table S1. Military Waste Stream Analysis (% by Weight). Table S2. Concentration (µg/g) of PAHs in the burn pit smoke PM. Table S3. Breathing parameters from the burn pit smoke exposures. Figure S1. Emission factors for VOCs of the burn pit smoke emissions. Figure S2. Mutagenicity dose–response curves of the smoldering samples in Salmonella strain TA98 with or without metabolic activation (S9). Figure S3. Mutagenicity dose–response curves of the flaming samples in Salmonella strains TA98 and TA100 with or without metabolic activation (S9). Figure S4. Correlation between biological responses and total PAH concentration of PM. [file 12989_2021_435_MOESM1_ESM.docx]

**Additional file 1 for**

**Chemistry, lung toxicity and mutagenicity of burn pit smoke-related particulate matter**

Yong Ho Kim^1, 2^, Sarah H. Warren^3^, Ingeborg Kooter^4^, Wanda Williams^2^, Ingrid J. George^5^, Samuel Vance^6^, Michael D. Hays^5^, Mark Higuchi^2^, Stephen H. Gavett^2^, David M. DeMarini^3^, Ilona Jaspers^1,7*^, M. Ian Gilmour^2*^

^1^Center for Environmental Medicine, Asthma and Lung Biology, University of North Carolina, Chapel Hill, NC 27599, USA

^2^Public Health and Integrated Toxicology Division, Center for Public Health and Environmental Assessment, U.S. Environmental Protection Agency, Research Triangle Park, NC 27711, USA

^3^Biomolecular and Computational Toxicology Division, Center for Computational Toxicology and Exposure, U.S. Environmental Protection Agency, Research Triangle Park, NC 27711, USA

^4^Department of Circular Economy and the Environment, The Netherlands Organisation of Applied Sciences, TNO, Utrecht, The Netherlands

^5^Air Methods and Characterization Division, Center for Environmental Measurements and Modeling, U.S. Environmental Protection Agency, Research Triangle Park, NC 27711, USA

^6^Oak Ridge Institute for Science and Education, Research Triangle Park, NC 27711, USA

^7^Department of Pediatrics, Department of Microbiology and Immunology, and Department of Environmental Sciences and Engineering, University of North Carolina, Chapel Hill, NC 27599, USA

^*^Correspondence: [ilona_jaspers@med.unc.edu](mailto:ilona_jaspers@med.unc.edu) and [gilmour.ian@epa.gov](mailto:gilmour.ian@epa.gov)

**Table S1.** *Military waste stream analysis (% by Weight)*

|  | **Paper** | **Plastic** | **Wood** | **Food** | **Liquid** | **Textile** | **Others** |
| --- | --- | --- | --- | --- | --- | --- | --- |
| Afghanistan #1 [[1](#_ENREF_1)] | 28 | 19 | 14 | 21 | 6 | 5 | 7 |
| Afghanistan #2 [[1](#_ENREF_1)] | 28 | 13 | 25 | 16 | 7 | 6 | 5 |
| Afghanistan #3 [[1](#_ENREF_1)] | 21 | 9 | 27 | 25 | 6 | 3 | 9 |
| Afghanistan #4 [[1](#_ENREF_1)] | 20 | 14 | 17 | 25 | 7 | 4 | 13 |
| Kuwait [[1](#_ENREF_1)] | 38 | 29 | 1 | 16 | 1 | 0 | 14 |
| Hawaii [[2](#_ENREF_2)] | 34 | 12 | 0 | 44 | 0 | 0 | 10 |
| DoD standard [[2](#_ENREF_2)] | 25 | 15 | 14 | 32 | 0 | 3 | 11 |

**Table S2.** *Concentration (µg/g) of PAHs in the burn pit smoke PM*

|  | | **Smoldering Smoke PM** | | | | | **Flaming Smoke PM** | | | | |
| --- | --- | --- | --- | --- | --- | --- | --- | --- | --- | --- | --- |
| **Class Component** | **Plywood** | | **Cardboard** | **Plastic** | **Mixture** | **Mixture**  **/Diesel** | **Plywood** | **Cardboard** | **Plastic** | **Mixture** | **Mixture**  **/Diesel** |
| 16 EPA PAHs |  | |  |  |  |  |  |  |  |  |  |
| Naphthalene | ND^a^ | | ND | 43 | 47 | 93 | 726 | 736 | 5,577 | 5,351 | 14,454 |
| Acenaphthylene | ND | | ND | ND | 5 | 5 | 249 | 280 | 3,325 | 1,911 | 10,063 |
| Acenaphthene | ND | | ND | 8 | ND | 29 | 16 | 26 | 178 | 145 | 683 |
| Fluorene | 5 | | 4 | 28 | 28 | 63 | 105 | 138 | 1,911 | 971 | 4,131 |
| Phenanthrene | 6 | | 9 | 43 | 70 | 80 | 1,297 | 1,524 | 7,817 | 5,391 | 13,155 |
| Anthracene | ND | | ND | ND | 7 | 7 | 124 | 166 | 1,467 | 743 | 3,303 |
| Fluoranthene | ND | | ND | 6 | 11 | 10 | 1,319 | 1,194 | 3,110 | 2,369 | 5,023 |
| Pyrene | ND | | ND | ND | 5 | 63 | 922 | 1,050 | 2,511 | 1,856 | 4,903 |
| Benz(a)anthracene | ND | | ND | ND | ND | ND | 285 | 338 | 873 | 549 | 1,648 |
| Chrysene | ND | | ND | 6 | 11 | 11 | 293 | 287 | 808 | 600 | 1,363 |
| Benzo(b)fluoranthene | ND | | ND | 3 | ND | ND | 486 | 416 | 1350 | 834 | 1,883 |
| Benzo(k)fluoranthene | ND | | ND | ND | ND | ND | 143 | 130 | 284 | 210 | 453 |
| Benzo(a)pyrene | ND | | ND | ND | ND | ND | 183 | 191 | 744 | 420 | 1,304 |
| Indeno[1,2,3-cd]pyrene | ND | | ND | ND | ND | ND | 150 | 137 | 468 | 286 | 650 |
| Dibenzo[ah]anthracene | ND | | ND | ND | ND | ND | 32 | 31 | 142 | 80 | 172 |
| Benzo[ghi]perylene | ND | | ND | ND | ND | ND | 139 | 152 | 591 | 321 | 777 |
| Oxy/Nitro-PAHs |  | |  |  |  |  |  |  |  |  |  |
| 1,4-Naphthoquinone | 6 | | 9 | 10 | 10 | 11 | 35 | 72 | 341 | 171 | 370 |
| 1-Naphthalenecarboxaldehyde | ND | | 4 | 8 | 6 | 6 | 246 | 306 | 677 | 456 | 1,229 |
| 9-Fluorenone | 10 | | 21 | 157 | 143 | 123 | 527 | 313 | 1,173 | 750 | 913 |
| 9,10-Anthraquinone | ND | | 5 | 7 | ND | 4 | 279 | 193 | 261 | 267 | 308 |
| 1,8-Naphthalic anhydride | ND | | ND | ND | ND | ND | 685 | 295 | 573 | 328 | 316 |
| Benzanthrone | ND | | ND | 4 | 4 | ND | 782 | 518 | 1,281 | 880 | 1650 |
| 1-Pyrenecarboxaldehyde | ND | | ND | ND | ND | ND | 39 | 29 | 50 | 39 | 60 |
| Benz[a]anthracene-7,12-quinone | ND | | ND | ND | ND | ND | 38 | 20 | 3 | 27 | 28 |
| 1-Nitronaphthalene | ND | | ND | ND | ND | ND | ND | ND | ND | ND | ND |
| 2-Nitronaphthalene | ND | | ND | ND | ND | ND | ND | ND | ND | ND | ND |
| 4-Nitrobiphenyl | ND | | ND | ND | ND | ND | 7 | 40 | 30 | 34 | 10 |
| 2-Nitrofluorene | ND | | ND | ND | ND | ND | ND | ND | ND | ND | ND |
| 9-Nitroanthracene | ND | | ND | ND | ND | ND | ND | ND | 148 | 35 | 52 |
| 3-Nitrofluoranthene | ND | | ND | ND | ND | ND | ND | ND | ND | ND | ND |
| 1-Nitropyrene | ND | | ND | ND | ND | ND | ND | ND | ND | ND | ND |
| 6-Nitrochrysene | ND | | ND | ND | ND | ND | ND | ND | ND | ND | ND |
| ∑ | 27 | | 52 | 323 | 347 | 505 | 9,107 | 8,582 | 35,693 | 25,024 | 68,901 |

^a^Values below the detection limit are listed as not detected (ND).

**Table S3.** *Breathing parameters from the burn pit smoke exposures*

|  | | **Smoldering** | | **Flaming** | | **Smoldering** | | **Flaming** | |
| --- | --- | --- | --- | --- | --- | --- | --- | --- | --- |
|  | | Pre | Post | Pre | Post | Pre | Post | Pre | Post |
|  | | **F (breaths/minute)** | | | | **MV (mL)** | | | |
| **4 h post exposure** | **Plywood** | 447.33 ± 25.46 | ***363.83 ± 53.54** | 405 ± 14.75 | 333.17 ± 20.28 | 109.17 ± 7.5 | 88.17 ± 11.25 | 108.67 ± 6.4 | 92.5 ± 6.53 |
|  | **Cardboard** | 463.33 ± 29.05 | ***374.33 ± 31.13** | 414.33 ± 9.51 | 357.83 ± 19.87 | 113 ± 6.83 | 93.83 ± 6.85 | 114.67 ± 3.95 | 96.83 ± 6.73 |
|  | **Plastic** | 406.33 ± 31.43 | 349 ± 35.09 | 434.17 ± 21.48 | 367.33 ± 19.59 | 96.17 ± 13.03 | 95.17 ± 14.43 | 112 ± 10.73 | 92.5 ± 3.57 |
|  | **Mixture** | 423 ± 22.8 | 366.17 ± 21.23 | 446 ± 19.83 | 388.83 ± 24.92 | 109.17 ± 5.66 | 99.33 ± 8.74 | 120.17 ± 6.85 | 100 ± 7.75 |
|  | **Saline** | 444.33 ± 26.31 | 392.83 ± 26.09 | 448.5 ± 18.25 | 387.17 ± 19.03 | 105.5 ± 7.54 | 113.83 ± 13.15 | 101.67 ± 5.21 | 83 ± 3.87 |
|  | **LPS** | 451 ± 12.81 | ***271.33 ± 35.1** | 432.67 ± 5.17 | ***258.33 ± 31.65** | 119.67 ± 4.98 | ***80.33 ± 7.28** | 102.33 ± 7.56 | ***71.33 ± 7.49** |
| **24 h post exposure** | **Plywood** | 445.67 ± 20.61 | 413.33 ± 29.03 | 417.67 ± 26.54 | 382.67 ± 15.06 | 99.5 ± 8.65 | 101 ± 6.94 | 94.17 ± 7.27 | 93.5 ± 4.09 |
|  | **Cardboard** | 444.33 ± 26.95 | 413.5 ± 30.59 | 435.5 ± 25.1 | 385.33 ± 20.64 | 103 ± 7.92 | 106.33 ± 9.85 | 100.17 ± 7.32 | 96 ± 7.38 |
|  | **Plastic** | 460 ± 27.27 | 440 ± 25.11 | 430.17 ± 19.79 | 403.17 ± 24.17 | 104.83 ± 8.18 | 109 ± 5.37 | 98 ± 9.76 | 97.5 ± 7.44 |
|  | **Mixture** | 449.5 ± 43.06 | 401.67 ± 30.84 | 465.33 ± 27.32 | ***384.33 ± 29.16** | 111.33 ± 14.93 | 103.83 ± 10.61 | 102.33 ± 7.81 | 94.5 ± 4.01 |
|  | **Saline** | 463.17 ± 27.53 | 412.33 ± 23.29 | 448.67 ± 24.1 | 398 ± 17.03 | 107.33 ± 10.79 | 104.17 ± 5.94 | 103.17 ± 7.11 | 95.5 ± 4.71 |
|  | **LPS** | 464 ± 25.59 | ***354.17 ± 22.33** | 480.17 ± 20.16 | ***381.17 ± 21.32** | 113.5 ± 8.71 | 92.17 ± 4.99 | 112.33 ± 6.15 | 109.17 ± 9.49 |
|  | | **Ti (msec)** | | | | **Te (msec)** | | | |
| **4 h post exposure** | **Plywood** | 53.17 ± 2.98 | 63.33 ± 6.77 | 57.67 ± 2.85 | 67 ± 3.92 | 104.5 ± 10.57 | ***167.17 ± 48.76** | 125.17 ± 11.65 | 156.17 ± 13.71 |
|  | **Cardboard** | 52.17 ± 6.17 | ***67.67 ± 9.62** | 57 ± 1.21 | 64.17 ± 2.63 | 98.33 ± 9.86 | 137 ± 14.78 | 110.33 ± 5.76 | 137.17 ± 10.87 |
|  | **Plastic** | 58.83 ± 3.3 | 69.33 ± 5.15 | 52.17 ± 3.44 | 59 ± 4.02 | 123.33 ± 12.77 | 142.5 ± 14.93 | 111.5 ± 9.76 | 136 ± 11.39 |
|  | **Mixture** | 54 ± 3.31 | 62.17 ± 3.52 | 52.83 ± 4.4 | 57.83 ± 4.69 | 122.5 ± 11.21 | 142 ± 9.68 | 105.5 ± 8.72 | 133.33 ± 12.05 |
|  | **Saline** | 51.33 ± 3.37 | 58.83 ± 3.76 | 51.17 ± 1.92 | 58.67 ± 2.95 | 114 ± 9.01 | 136.17 ± 11.06 | 103.5 ± 7.07 | 137.67 ± 10.56 |
|  | **LPS** | 53.5 ± 2.51 | ***76.33 ± 6.48** | 52.5 ± 2.23 | ***68 ± 4.82** | 100.83 ± 5.39 | ***209.83 ± 37.3** | 111.33 ± 3.68 | ***223.5 ± 24.41** |
| **24 h post exposure** | **Plywood** | 51 ± 2.59 | 54.33 ± 3.91 | 55 ± 4.55 | 56.83 ± 2.5 | 107.33 ± 8.71 | 133 ± 16.54 | 127.83 ± 17.16 | 135.17 ± 7.35 |
|  | **Cardboard** | 49.67 ± 2.73 | 56.67 ± 4.67 | 49.67 ± 2.92 | 58.5 ± 4.84 | 119.33 ± 13.84 | 119.5 ± 13.24 | 123.33 ± 17.32 | 139.33 ± 15.02 |
|  | **Plastic** | 50.83 ± 3.24 | 52.5 ± 3.55 | 54.5 ± 3.65 | 57.5 ± 4.65 | 101 ± 8.77 | 109.83 ± 9.62 | 117 ± 12.6 | 128.83 ± 12.16 |
|  | **Mixture** | 53.5 ± 6.27 | 59 ± 5.14 | 48.83 ± 3.31 | 58.5 ± 5.11 | 114.33 ± 22.86 | 124.67 ± 15.45 | 105.17 ± 12.3 | 138 ± 17.12 |
|  | **Saline** | 49 ± 3.63 | 54.33 ± 3.6 | 52.17 ± 4.56 | 57.5 ± 4.1 | 111.17 ± 16.57 | 129.67 ± 11.86 | 113.33 ± 17 | 127.83 ± 7.13 |
|  | **LPS** | 51.17 ± 2.9 | 55.83 ± 1.68 | 46.5 ± 2.64 | 51.17 ± 1.99 | 109.83 ± 14.25 | 151 ± 17.73 | 97.33 ± 4.33 | 132.33 ± 11.07 |
|  | | **PIF (mL/sec)** | | | | **PEF (mL/sec)** | | | |
| **4 h post exposure** | **Plywood** | 8.34 ± 0.49 | 7.82 ± 0.81 | 8.33 ± 0.49 | 7.7 ± 0.48 | 5.57 ± 0.26 | 5.34 ± 0.68 | 5.47 ± 0.39 | 4.52 ± 0.42 |
|  | **Cardboard** | 8.61 ± 0.57 | 7.52 ± 0.64 | 8.35 ± 0.33 | 7.54 ± 0.46 | 5.64 ± 0.28 | 4.7 ± 0.29 | 5.72 ± 0.3 | 4.57 ± 0.31 |
|  | **Plastic** | 7.47 ± 0.87 | 7.45 ± 0.98 | 8.68 ± 0.75 | 7.84 ± 0.48 | 4.74 ± 0.65 | 4.95 ± 0.74 | 5.59 ± 0.55 | 4.69 ± 0.37 |
|  | **Mixture** | 8.64 ± 0.52 | 8.03 ± 0.72 | 9.09 ± 0.48 | 8.21 ± 0.59 | 5.39 ± 0.28 | 4.79 ± 0.4 | 5.82 ± 0.36 | 4.87 ± 0.35 |
|  | **Saline** | 8.45 ± 0.6 | 8.49 ± 0.56 | 7.85 ± 0.43 | 6.78 ± 0.34 | 5.44 ± 0.39 | 6.57 ± 1.53 | 4.94 ± 0.31 | 3.91 ± 0.15 |
|  | **LPS** | 8.93 ± 0.37 | 7.52 ± 0.39 | 8.01 ± 0.69 | 7.89 ± 0.73 | 6.11 ± 0.3 | 6.45 ± 0.84 | 5.01 ± 0.3 | 6.28 ± 0.58 |
| **24 h post exposure** | **Plywood** | 8.15 ± 0.57 | 8.51 ± 0.44 | 7.67 ± 0.53 | 7.92 ± 0.36 | 5.26 ± 0.43 | 5.09 ± 0.41 | 4.82 ± 0.38 | 4.41 ± 0.16 |
|  | **Cardboard** | 8.38 ± 0.45 | 8.22 ± 0.6 | 8.52 ± 0.48 | 8.13 ± 0.55 | 5.05 ± 0.31 | 4.98 ± 0.47 | 4.84 ± 0.4 | 4.56 ± 0.43 |
|  | **Plastic** | 8.06 ± 0.46 | 8.45 ± 0.34 | 7.91 ± 0.85 | 8.03 ± 0.74 | 5.21 ± 0.33 | 5.13 ± 0.25 | 5.07 ± 0.54 | 4.77 ± 0.37 |
|  | **Mixture** | 8.56 ± 0.92 | 7.98 ± 0.72 | 8.16 ± 0.57 | 7.79 ± 0.23 | 5.47 ± 0.81 | 5.04 ± 0.57 | 5.03 ± 0.36 | 4.48 ± 0.2 |
|  | **Saline** | 8.59 ± 0.67 | 8.46 ± 0.47 | 8.37 ± 0.4 | 7.77 ± 0.34 | 5.47 ± 0.54 | 5.24 ± 0.35 | 5.12 ± 0.33 | 4.59 ± 0.22 |
|  | **LPS** | 8.74 ± 0.41 | 8.51 ± 0.37 | 8.87 ± 0.43 | 9.98 ± 0.57 | 5.81 ± 0.48 | 5.79 ± 0.44 | 5.52 ± 0.35 | 6.26 ± 0.56 |
|  | | **RT (msec)** | | | | **TV (mL)** | | | |
| **4 h post exposure** | **Plywood** | 0.52 ± 0.03 | 0.87 ± 0.35 | 0.5 ± 0.04 | 0.54 ± 0.03 | 0.25 ± 0.01 | 0.27 ± 0.03 | 0.28 ± 0.01 | 0.29 ± 0.01 |
|  | **Cardboard** | 0.53 ± 0.05 | 0.56 ± 0.04 | 0.51 ± 0.02 | 0.47 ± 0.07 | 0.25 ± 0.01 | 0.26 ± 0.01 | 0.29 ± 0.01 | 0.28 ± 0.01 |
|  | **Plastic** | 0.48 ± 0.02 | 0.66 ± 0.05 | 0.47 ± 0.03 | 0.57 ± 0.08 | 0.25 ± 0.01 | 0.28 ± 0.01 | 0.27 ± 0.02 | 0.26 ± 0.01 |
|  | **Mixture** | 0.46 ± 0.03 | 0.49 ± 0.03 | 0.47 ± 0.03 | 0.47 ± 0.02 | 0.27 ± 0.01 | 0.28 ± 0.01 | 0.28 ± 0.01 | 0.27 ± 0.01 |
|  | **Saline** | 0.49 ± 0.03 | 0.45 ± 0.03 | 0.48 ± 0.02 | 0.44 ± 0.01 | 0.25 ± 0.01 | 0.39 ± 0.11 | 0.23 ± 0.01 | 0.23 ± 0.01 |
|  | **LPS** | 0.54 ± 0.06 | 2.26 ± 0.75 | 0.47 ± 0.06 | 2.49 ± 0.51 | 0.28 ± 0.01 | 0.32 ± 0.03 | 0.25 ± 0.02 | 0.29 ± 0.02 |
| **24 h post exposure** | **Plywood** | 0.55 ± 0.05 | 0.46 ± 0.04 | 0.51 ± 0.03 | 0.43 ± 0.04 | 0.23 ± 0.01 | 0.26 ± 0.01 | 0.24 ± 0.01 | 0.26 ± 0.01 |
|  | **Cardboard** | 0.43 ± 0.03 | 0.43 ± 0.02 | 0.42 ± 0.03 | 0.41 ± 0.04 | 0.24 ± 0.01 | 0.27 ± 0.01 | 0.24 ± 0.01 | 0.26 ± 0.01 |
|  | **Plastic** | 0.49 ± 0.02 | 0.43 ± 0.02 | 0.55 ± 0.03 | 0.48 ± 0.03 | 0.24 ± 0.02 | 0.26 ± 0.01 | 0.24 ± 0.01 | 0.25 ± 0.02 |
|  | **Mixture** | 0.46 ± 0.04 | 0.46 ± 0.05 | 0.46 ± 0.03 | 0.42 ± 0.03 | 0.25 ± 0.01 | 0.27 ± 0.01 | 0.23 ± 0.02 | 0.26 ± 0.02 |
|  | **Saline** | 0.49 ± 0.02 | 0.49 ± 0.04 | 0.46 ± 0.04 | 0.43 ± 0.03 | 0.24 ± 0.01 | 0.27 ± 0.01 | 0.24 ± 0.01 | 0.26 ± 0.01 |
|  | **LPS** | 0.52 ± 0.03 | 1.02 ± 0.13 | 0.47 ± 0.04 | 0.93 ± 0.13 | 0.25 ± 0.01 | 0.28 ± 0.01 | 0.24 ± 0.01 | 0.29 ± 0.02 |

 *Values are statistically significant compared to pre-exposure values (*p* < 0.05) n= 6 mice per group.


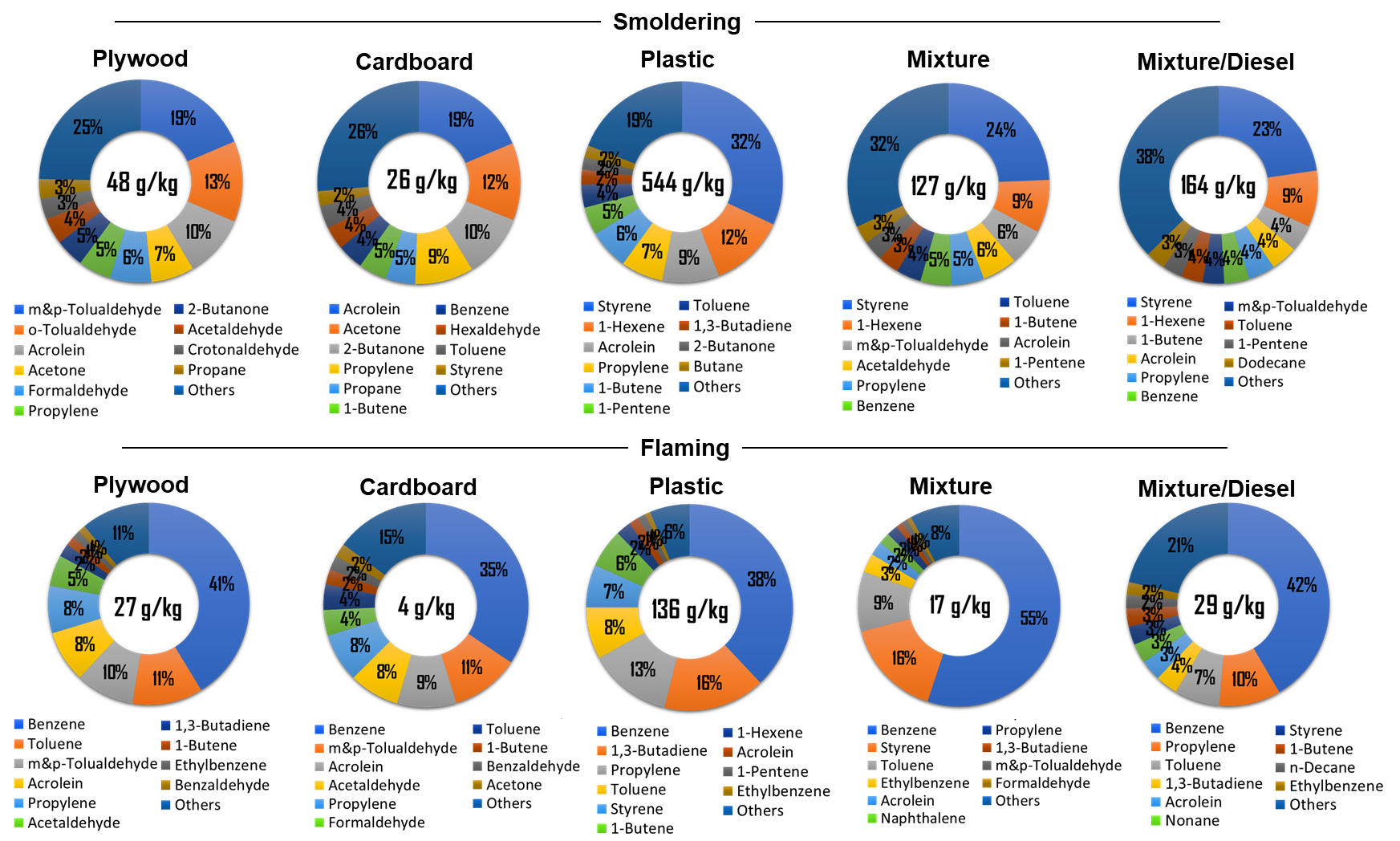


**Figure S1.** *Emission factors for VOCs of the burn pit smoke emissions.*


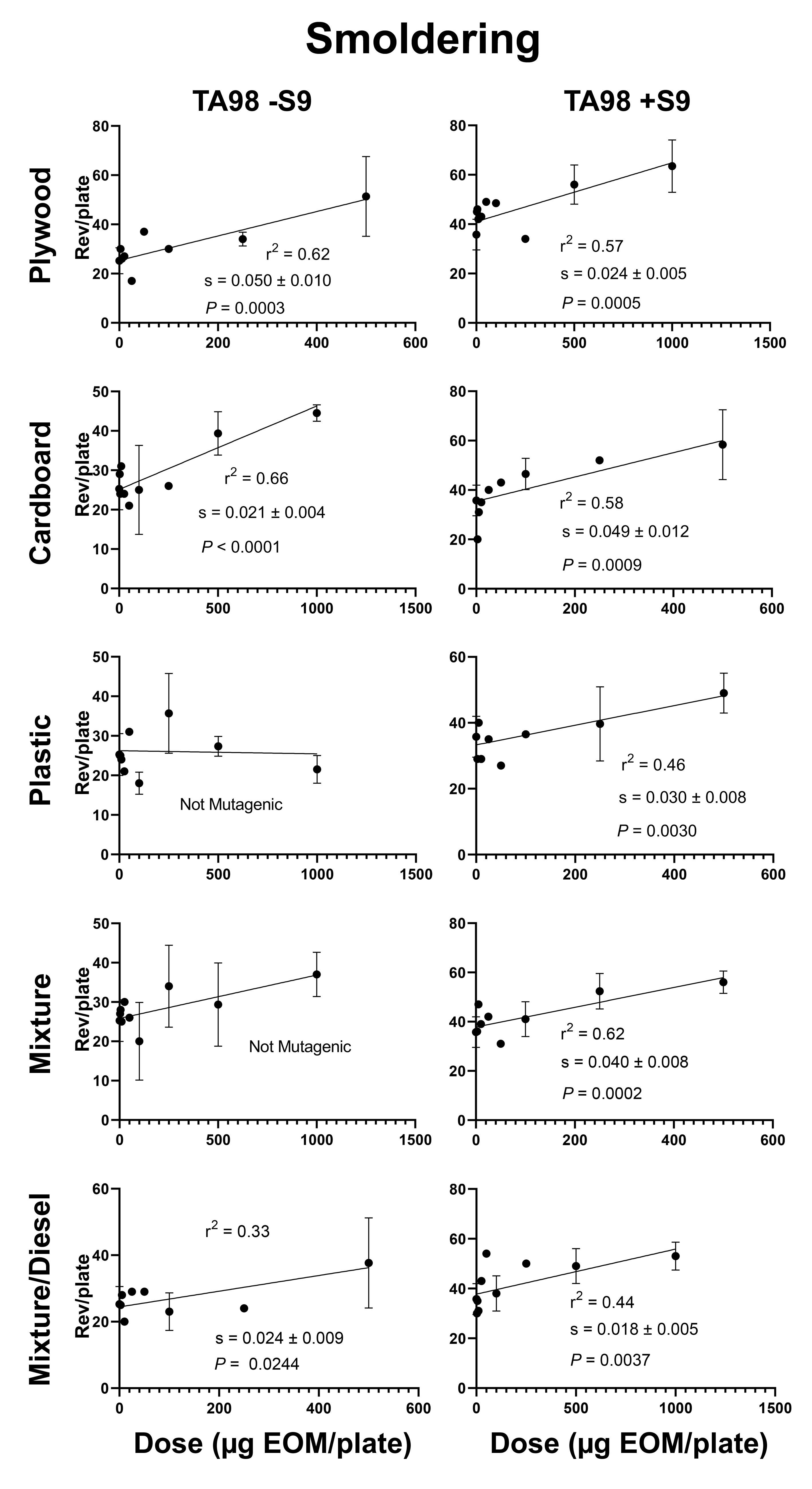


**Figure S2.** *Mutagenicity dose-response curves of the smoldering samples in Salmonella strain TA98 with or without metabolic activation (S9).*  These curves were constructed by combining data from 4 independent experiments, each at 1 plate/dose. Slopes (s) of the linear regressions over the initial linear portion of the curves are the mutagenic potencies (rev/µg EOM) ± SEM. Samples with *P* < 0.05 based on a trend test were considered mutagenic; samples with *P* > 0.05 were considered non-mutagenic and assigned mutagenic potencies of zero.


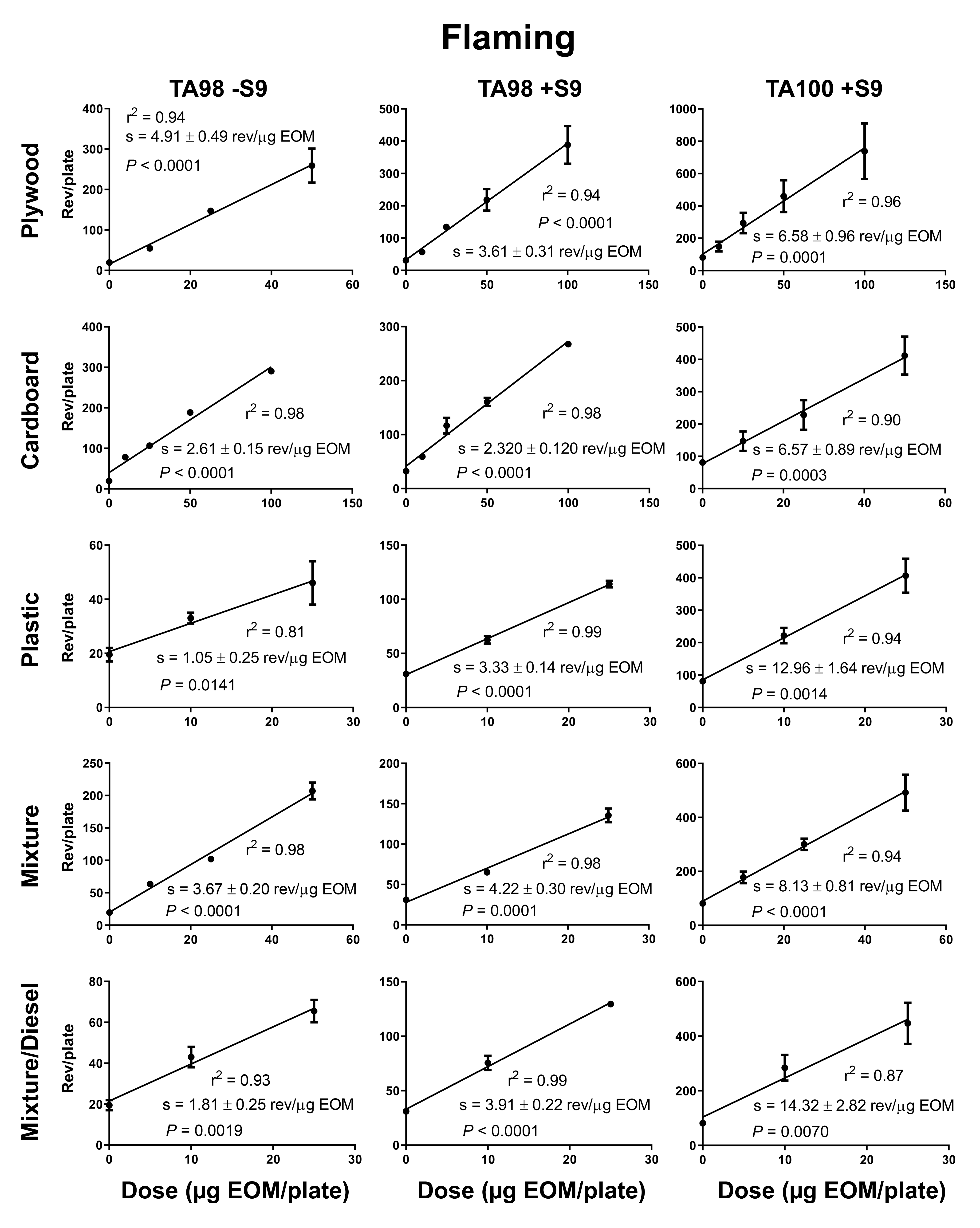


**Figure S3.** *Mutagenicity dose-response curves of the flaming samples in Salmonella strains TA98 and TA100 with or without metabolic activation (S9).*  These curves were constructed by combining data from 2 independent experiments, each at 1 plate/dose. Slopes (s) of the linear regressions over the initial linear portion of the curves are the mutagenic potencies (rev/µg EOM) ± SEM. Samples with *P* < 0.05 based on a trend test were considered mutagenic.


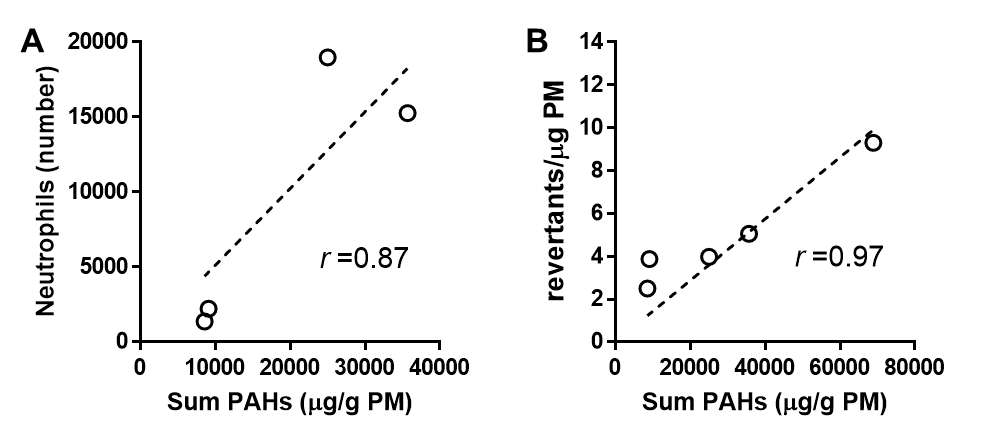


**Figure S4**. *Correlation between biological responses and total PAH concentration of PM*. **a** lung toxicity vs. total PAHs, **b** mutagenicity vs. total PAHs. Lung toxicity (neutrophil numbers) and mutagenicity (rev/µg PM in TA100 +S9) data were obtained from **Figure 4** and **Table 2**, respectively. Total sum of PAHs was obtained from **Table S2**. *r* = Pearson correlation coefficient.

**References**

1. USALIA. US Army Central (USARCENT) Area of Responsibility (AOR) Contigency Base Waste Stream Analysis 2013.

2. Aurell J, Barnes M, Gullett BK, Holder A, Eninger R. Methodology for characterizing emissions from small (0.5–2 MTD) batch-fed gasification systems using multiple waste compositions. Waste Management. 2019;87:398-406.
